# Humanization of CD47 enables development of functional human neutrophils via postirradiation remodeling of the bone marrow

**DOI:** 10.1073/pnas.2426546122

**Published:** 2025-09-16

**Authors:** Esen Sefik, William Philbrick, Fengrui Zhang, Kriti Agrawal, Brian Van Lee, Johannes Sam, Kutay Karatepe, Yunjiang Zheng, Kaixin Liang, Sophia Peng, Haris Mirza, Athreya Rangavajhula, Perrine Simon, Neha Arun, Priyanka Babu, Elizabeth Eynon, Michael Chiorazzi, Liang Shan, Stephanie Halene, Hongbo R. Luo, Anthony Rongvaux, Yuval Kluger, Richard A. Flavell

**Affiliations:** ^a^Department of Immunobiology, Yale School of Medicine, New Haven, CT 06519; ^b^Department of Internal Medicine, Section of Endocrinology, Yale School of Medicine, New Haven, CT 06519; ^c^Computational Biology & Biomedical Informatics Program, Yale University, New Haven, CT 06519; ^d^Roche Innovation Center Zurich, Schlieren 8952, Switzerland; ^e^Department of Cell Biology, Yale School of Medicine, New Haven, CT 06519; ^f^Department of Pathology, Yale School of Medicine, New Haven, CT 06519; ^g^Department of Microbial Pathogenesis, Yale School of Medicine, New Haven, CT 06519; ^h^Division of Infectious Diseases, Department of Medicine, Washington University School of Medicine in St. Louis, St. Louis, MO 63110; ^i^Department of Internal Medicine, Yale Comprehensive Cancer Center, Yale University School of Medicine, New Haven, CT 06519; ^j^Department of Pathology, Brigham and Women’s Hospital, Boston, MA 02115; ^k^Translational Science and Therapeutics Division, Fred Hutchinson Cancer Center, Seattle, WA 98109; ^l^Program in Applied Mathematics, Yale University, New Haven, CT 06511; ^m^HHMI, Yale University School of Medicine, New Haven, CT 06519

**Keywords:** immunobiology, immunology, neutrophils, humanized mice, CD47

## Abstract

Murine and human immune systems differ significantly, particularly within the myeloid lineage. Humanized mice, generated by transplanting human hematopoietic stem, progenitor cells into genetically modified mice, are invaluable to study human immune development and function in vivo. However, a major limitation of current models is suboptimal myelopoiesis, particularly lack of functional human neutrophils, hampering the modeling of human immune responses and chronic diseases. Here, we describe a humanized mouse model, named MaGIC for genes replaced, in the *C57Bl/6 N* strain, which improves human myelopoiesis and enables development of functional human neutrophils. In MaGIC mice, human cytokines M-CSF/CSF1(M), GM-CSF/CSF2(G) and IL-6(I) are knocked-in replacing mouse genes and murine *IL2rg and Rag1*(a) are deleted. Human THPO in these mice supports human hematopoiesis. More importantly, insertion of human CD47 (C) under the control of endogenous mouse CD47 promoter enables xenotransplantation and human neutrophil development. MaGIC mice support all human neutrophil subsets found in human bone marrow and blood, a major improvement. This is achieved by creating a niche postirradiation for human granulocyte–macrophage progenitors via reduced murine CD47 and physiological levels of human CD47. These mice also have mature human monocytes, tissue macrophages, alveolar macrophages, dendritic cells, and NK cells, enabled by humanized M-CSF and GM-CSF. Human neutrophils in MaGIC mice are fully functional in chemotaxis, phagocytosis, reactive oxygen species production, and neutrophil extracellular trap formation in response to inflammation. MaGIC mice address critical gaps in current models and enable incisive translational research on human neutrophils, advancing studies in infectious, autoimmune, and inflammatory diseases.

## Significance

Mice are essential for studying immune responses, but functional differences between human and murine systems limit their utility, particularly for neutrophils. Humanized mice with a human immune system have advanced understanding of human immunity, yet suboptimal myelopoiesis and lack of functional neutrophils remain challenges. We developed MaGIC mice, a model expressing human CD47, M-CSF, GM-CSF, IL-6, and thrombopoietin while reducing murine CD47 on the C57Bl/6 N background. MaGIC mice support robust human hematopoiesis, including functional neutrophils, monocytes, and tissue macrophages. These neutrophils, phenotypically and developmentally comparable to human neutrophils, exhibit chemotaxis, phagocytosis, reactive oxygen species production, and neutrophil extracellular trap formation in response to inflammatory stimuli, providing an innovative platform to study human neutrophils in infectious, autoimmune, and inflammatory diseases.

Mice are invaluable models for studying in vivo immune responses. Despite the structural and compositional similarities between murine and human immune systems, millions of years of evolutionary divergence have led to notable differences. While human and mouse cells share many lineage-defining markers and aspects of cellular identity, their functions, activities, and effector or regulatory mechanisms often differ significantly. Neutrophils display species-specific differences between mice and humans in their abundance, lifespan, and activation, leading to distinct impacts on immunity and disease pathogenesis (1, 2).

Neutrophils play a vital role in responding to injury and infection and are now increasingly recognized for their contribution to homeostatic immune surveillance in both humans and mice (3–6). While human neutrophils, with a lifespan of approximately 5 d, are the most abundant circulating immune cells (comprising 50 to 70% of leukocytes), mouse neutrophils have a much shorter lifespan (around 1 d) and represent a smaller fraction of circulating leukocytes (10 to 25%) (2, 7). Both human and mouse neutrophils play vital roles in defending against microbial infections through multiple mechanisms, including phagocytosis, the release of reactive oxygen species (ROS), antimicrobial peptides and proteases, production of cytokines and chemokines that activate and attract other immune cells, and their unique ability to form neutrophil extracellular traps (NETs) (1, 6, 8–17). While these core functions are shared among mice and humans, significant differences exist in their maturation, migration, stimulation, and function across species (1, 9–17). Human and mouse neutrophils exhibit differences in the expression and binding of selectins [such as L-selectin (9)] and chemokine receptors [e.g., CXCR1 (18) expression in human neutrophils, IL-8 as a human neutrophil chemoattractant (19)], which influence their migratory capacity. They also differ in Fc receptor expression (e.g., FcαRI is present only in humans) (12), which mediate their killing ability, in cytokine production (IL-10 is produced only by mouse neutrophils) (14), in ligand responsiveness that activates ROS (20) and in patterns of defensins in granules (mouse neutrophils lack alpha defensins) (15), all of which mediate the inflammatory response. Current studies also highlighted heterogeneity in neutrophils in circulation and tissues. In the context of cancer alone, there have been at least seven classes of neutrophils with distinct molecular signatures identified in humans, some of which do not overlap with mouse studies (1, 21–24). These interspecies differences in the overall biology of neutrophils underline the need for tractable models that support human neutrophils and have important implications for various pathologies.

Humanized mice bearing a functional human immune system generated by transplantation of human hematopoietic stem and progenitor cells (HSPCs) into genetically modified mice serve as an invaluable tool to study the development and function of the human immune system in vivo (25). A major technological limitation of most current humanized mouse models is suboptimal myelopoiesis, and this lack of functional myeloid cells, particularly of human neutrophils, prevents modeling of key components of the human immune response and immune cell driven pathologies in chronic diseases. Targeting the G-CSF cytokine receptor axis has partially improved human neutrophil levels in circulation and tissues (26). However, these models rely on the depletion of mouse neutrophils to restore human neutrophils, rendering the mice highly susceptible to infections and invasion by pathobionts. This susceptibility reduces breeding efficiency and poses significant challenges in animal husbandry and the study of inflammatory diseases.

In this study, we describe a humanized mouse model named MaGIC in the *C57Bl/6 N* strain that improves human myelopoiesis and enables the development of functional human neutrophils. MaGIC mice (acronym for genes modified) express the human MCSF(CSF1) (M), human GM-CSF (G), human thrombopoietin, human IL-6 (I) and human CD47 (C) are deficient for mouse *IL2rg and Rag1* (a). In addition to human neutrophils provided by the humanization of CD47 and the disruption of mouse CD47, the expression of human M-CSF, THPO, GM-CSF, and IL-6 in MaGIC mice establishes a highly efficient model of human hematopoiesis, enabling the development of all subsets of monocytes, tissue macrophages, and alveolar macrophages (25). Gene modifications and their associated outcome are summarized in *SI Appendix*, Table S1.

## Results

### Humanization of CD47 in Mice Yields More Human Myeloid Cells Compared to Humanization of SIRPA.

**Fig. 1.**
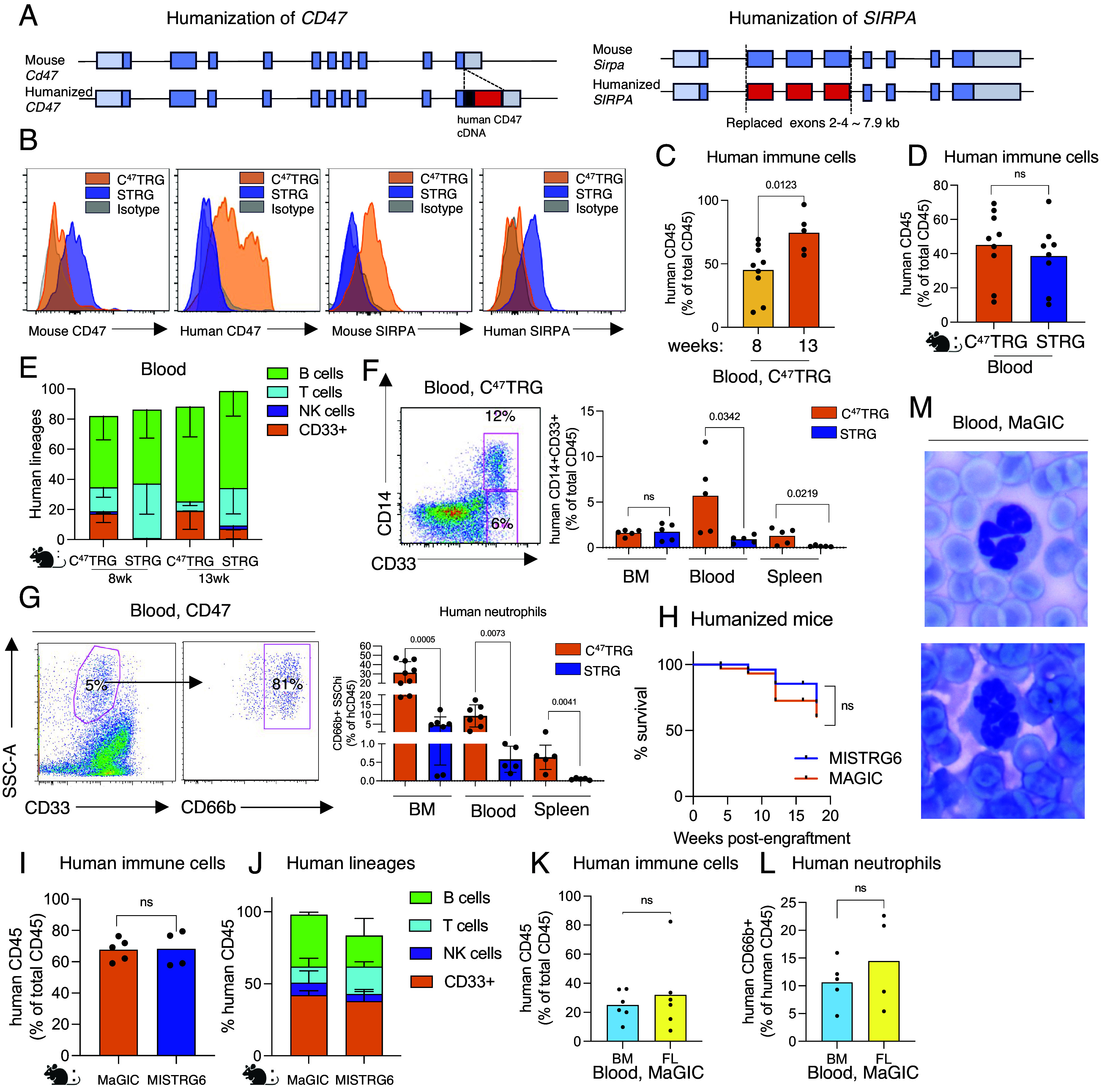
Humanization of CD47 supports human neutrophils. (*A*) *Top*: CD47 humanization: an internal ribosomal entry site (IRES) (black rectangle) and a human *CD47* complete coding (cDNA) sequence (adjoining red rectangle) of ~1 kb have been inserted immediately downstream of stop codon in the last exon of the mouse *Cd47* gene via CRISPR-mediated, homology-directed integration. This design preserves both upstream and intronic regulatory elements governing expression of the mouse gene. *Bottom*: Humanization of the mouse *Sirpa* gene: the replacement of key genomic sequences was carried out by homologous recombination in C57BL/6 ES cells. Exons 2 to 4 of the mouse *Sirpa* gene (*Top*) have been replaced with the corresponding exons (coding sequences in red) from the human SIRPA gene (*Bottom*). A 7.9 kb segment has been integrated into the mouse gene so that the encoded chimeric protein has the mouse signal sequence (mouse exon 1) followed by the entire human extracellular region corresponding to human amino acids 28 to 362 (human exons 2 to 4) fused to the intracellular portion of the mouse SIRPα protein (mouse exons 5 to 8) for proper signaling in mouse cells. (*B*) Representative flow cytometry staining of human and mouse CD47 and SIRPA in blood of C^47^TRG and STRG mice. C^47^TRG mice express human CD47 at physiological levels have significantly reduced levels of mouse CD47. STRG mice express human SIRPA and lack expression of mouse SIRPA. These mice are *on a Rag1^−/−^/Il2rg^−/−^* background and encode human THPO. (*C*) Humanization measured as proportions of human immune cells (human CD45+) among all immune cells (mouse or human CD45+ combined) at different time points in C^47^TRG mice with humanized CD47. Mice were irradiated (~320 cGy) and engrafted with 150,000 human CD34+ cells, which include HSPCs. Humanization is reported at 8 or 13 wk of engraftment. Mean values are shown with data points. Statistical analysis was performed using the paired two-tailed *t* test. (*D*) Humanization in C^47^TRG (humanized CD47) and STRG (humanized SIRPA) mice with or SIRPA measured as proportion of human immune cells (human CD45+) among all immune cells (mouse or human CD45+). Mice were irradiated and engrafted with human CD34+ cells which include HSPCs. Mean values are shown with data points. Statistical analysis was performed using the unpaired two-tailed *t* test. (*E*) Human immune cell lineages in peripheral blood of reconstituted C^47^TRG or STRG humanized mice at 8 and 13 wk postengraftment. Expression of CD19, CD3, NKP46, and CD33 marks B, T, NK, and myeloid cell lineages in blood, respectively. (*F*) *Left*: representative flow cytometry plot of human CD33+ CD14+ monocytes within the human CD45+ population in C^47^TRG mice. *Right*: Frequencies of human monocytes in BM, spleen, and blood of C^47^TRG and STRG mice. (*G*) *Left*: Representative flow cytometry plot showing CD66b+ SSChi+ CD33lo cells in blood of C^47^TRG mice. *Right*: Frequencies of human neutrophils BM, blood, liver, lungs, and spleen of C^47^TRG and STRG mice. Mean values are shown with data points. Statistical analysis was performed using the unpaired two-tailed *t* test. (*H*) Survival curve of reconstituted MaGIC (CD47 humanized) and MISTRGv2 (SIRPA humanized) mice postengraftment. N = 8. Mean values are shown with data points. Statistical analysis was performed using the unpaired two-tailed *t* test. (*I*) Humanization measured as proportion of human immune cells among mouse and human immune cells in blood of engrafted MaGIC and MISTRGv2 mice. Mice were irradiated (~250 cGy) and engrafted with 150,000 human CD34+ cells, which include HSPCs. Humanization is reported at 15 wk of engraftment. Mean values are shown with data points. Statistical analysis was performed using the unpaired two-tailed *t* test. (*J*) Human immune cell lineages in blood of engrafted MaGIC and MISTRGv2 mice. (*K*) Humanization in MaGIC mice engrafted with human CD34+ derived from adult BM or fetal liver (FL). Mice were engrafted with 200,000 BM-derived and 80,000 FL-derived CD34+ cells. Humanization is reported at 18 wk of engraftment. Mean values are shown with data points. Statistical analysis was performed using the unpaired two-tailed *t* test. (*L*) Frequencies of blood human neutrophils marked by CD66b expression in MaGIC mice engrafted with human CD34+ derived from adult BM or FL. Mice were irradiated (~250 cGy) and were engrafted with 200,000 BM-derived and 80,000 FL- derived CD34+ cells. Mean values are shown with data points. Statistical analysis was performed using the unpaired two-tailed *t* test. (*M*) Giemsa staining of engrafted MaGIC blood smears. Representative of N = 4 mice. All data are representative of at least two independent experiments.

To generate mice expressing human CD47 on *a Rag1^−/−^/Il2rg^−/−^* background, we inserted a human *CD47* coding segment into the last exon of the mouse *Cd47* gene by CRISPR-mediated, homology-dependent integration (Fig. 1*A* and *SI Appendix*, *SI Materials and Methods*, Fig. S1 *A*–*C*, and Table S1). This approach allowed human CD47 to be expressed at physiological levels but resulted in significantly reduced levels of mouse CD47 (Fig. 1*B* and *SI Appendix*, Fig. S1*D*). In these mice, we also incorporated human thrombopoietin (THPO) by inserting a complete coding (cDNA) sequence of human *THPO* (~1 kb) into the 5′ UTR of the mouse *Thpo* gene, resulting in a mouse that expresses human THPO and supports human hematopoiesis (*SI Appendix*, *SI Materials and Methods*, Fig. S1*C*, and Table S1). These mice were also on *a Rag1^−/−^/Il2rg^−/−^* background in the *C57Bl/6 N* strain (*SI Appendix*, Fig. S1 *A*–*C*). We refer to these mice as C^47^TRG, an acronym for genes modified (*CD47^h/h^, THPO^h/h^, Rag1^−/−^*, and *Il2rg^−/−^*). Mice that express human SIRPA were generated by homologous recombination in C57BL/6 embryonic stem (ES) cells, replacing exons 2 to 4 of the mouse *Sirpa* gene with the corresponding exons from the human SIRPA gene (Fig. 1*A* and *SI Appendix*, Fig. S1 *A*–*C*). We refer to these mice as STRG *(SIRPA ^h/h^, THPO ^h/h^, Rag1^−/−^*, and *Il2rg^−/−^*). In STRG mice, there are no detectable levels of mouse SIRPA protein, which is replaced by physiological expression of human SIRPA protein (Fig. 1*B*). In order to facilitate transplantation of human immune cells and maximize survival, we optimized the irradiation dose for each mouse strain, balancing lethality due to irradiation induced toxicity and humanization, measured as the proportion of human immune cells among total immune cells. Humanization was highly efficient in mice with humanized CD47 or SIRPA and supported multiple human lineages (Fig. 1 *C*–*E* and *SI Appendix*, Fig. S1 *E*, *F* and *G*). Perturbation and humanization of CD47–SIRPA axis was necessary for human immune cell engraftment (*SI Appendix*, Fig. S1*E*). In the STRG mice, heterozygous expression of human SIRPA is sufficient to support human immune cell engraftment (*SI Appendix*, Fig. S1*E*). Human lineages in blood and tissues were comparable for T and B cells in both C^47^TRG, and STRG mice, but C^47^TRG mice had higher proportions of myeloid cells (marked by CD33) (Fig. 1*E* and *SI Appendix*, Fig. S1*G*). This partial advantage in the myeloid lineage of C^47^TRG was reflected in more CD33+ CD14+ monocytes in blood in C^47^TRG compared with STRG (Fig. 1*F*). Interestingly, when analyzing CD33+ cells, we identified a population of cells that had lower expression of CD33 but were high in the side scatter (SSC) parameter, a property reminiscent of human neutrophils (Fig. 1*G* and *SI Appendix*, Fig. S1 *H* and *I*). Further characterization of this population with additional markers such as CD66b revealed that these CD33+ cells were indeed human neutrophils (Fig. 1*G*, *SI Appendix*, Fig. S1 *H* and *I*). Human neutrophils were largely absent in mice harboring human SIRPA, but were abundant in bone marrow (BM) and blood of mice with humanized CD47 (Fig. 1*G* and *SI Appendix*, Fig. S1 *H* and *I*). This was a very exciting finding because a major limitation of current humanized mouse models is the lack of mature and functional human neutrophils in circulation. Our published work studying the GCSF–GCSFR axis (26) allowed depletion of mouse neutrophils which partially restored human neutrophils, but we appreciated that humanization of CD47 may be the key in improving humanized models that support human neutrophils, the most abundant leukocyte in humans corresponding to half of the immune cells in blood.

### Generation of MaGIC and MISTRGv2 Mice by Humanizing of CSF1, CSF2, and IL6.

Developing a humanized mouse with a comprehensive human immune system requires recapitulating both myeloid and lymphoid lineages (25). Although modification of the SIRPA–CD47 axis facilitated xenotransplantation, additional factors were necessary for development of all subsets of monocytes, tissue macrophages, and NK cells (25, 27–32). To accomplish this, we incorporated human CSF1 (M-CSF,M), CSF2 (GM-CSF,G), IL6 (I) into C^47^TRG mice and STRG mice, creating MaGIC and MISTRGv2 mice (*SI Appendix*, *SI Materials and Methods*, Figs. S2–S4, and Table S1). To avoid confusion with the earlier MISTRG mouse developed on the BALB/c background (27, 29, 33), we refer to the new version on the C57BL/6 background as MISTRGv2.

#### Humanization of CSF1(M-CSF).

In humans, three subsets of monocytes have been phenotypically and functionally described based on differential expression of CD14 and CD16 (34). In our earlier work we have shown that CD16+CD14^dim^ monocytes largely rely on human M-CSF encoded by human *CSF1* gene (29, 30), which we incorporated. In line with our published results (30), monocytes in C^47^TRG were predominantly CD14+ (Fig. 1*F*) and still lacked expression of CD16, suggesting that human CSF1 is still required to induce physiological maturation of all monocyte subsets found in human blood.

We generated *CSF1* knock-in mice by inserting a human *CSF1* coding region into the second exon of the mouse *Csf1* gene (*SI Appendix*, Fig. S2*A*), creating a mouse/human hybrid coding region. This design also includes a truncated human 3′ UTR preserving several identified functional elements responsible for regulating *CSF1* mRNA turnover (35). In addition, the mouse promoter, exon 1, and intron 1 are left intact, so that splicing reconstitutes the complete coding sequence. Transcription termination immediately following the human coding region is designed to prevent transcription of the downstream portion of the mouse *CSF1* gene and thus eliminate nonsense-mediated decay of the humanized mRNA. We validated expression of the human *CSF1* transcript and measured CSF1(M-CSF) protein in serum by ELISA (*SI Appendix*, Fig. S2*B*). CSF1 protein in serum was present at comparable levels to humans. Humanization of M-CSF in C^47^TRG mice enabled full maturation of blood monocytes, development of human tissue macrophages (*SI Appendix*, Fig. S2 *C*–*E*). These myeloid cells are known to produce human IL-15, a cytokine necessary for development of human NK cells (29). This myeloid-derived IL-15 also therefore enabled development of human NK cells(*SI Appendix*, Fig. S2*F*). These human NK cells also showed features of tissue residency and activation (*SI Appendix*, Fig. S2*F*).

#### Humanization of CSF2 (GM-CSF).

Next, we incorporated human CSF2, also known as GM-CSF, which is at least necessary for development of alveolar macrophages (25, 28, 32). Mice expressing human CSF2 were generated by CRISPR-mediated gene replacement, essentially substituting the entire human transcriptional unit for the mouse paralog (*SI Appendix*, Fig. S3*A*). Using a CRISPR-mediated, targeted integration, mouse *Csf2* gene (~2 kb) was excised and the similarly sized human *CSF2* homolog was integrated, effectively placing the human transcriptional unit under the control of the mouse promoter and upstream regulatory elements. We validated expression of the human *CSF2* transcript and protein in the homogenized lung tissue (*SI Appendix*, Fig. S3*B*). Upon LPS stimulation, CSF2 levels were increased in mice encoding human *CSF2,* supporting physiological regulation of CSF2 during inflammation (*SI Appendix*, Fig. S3*B*). Human CSF2 is essential for supporting human alveolar macrophages in lungs (25, 28, 32). As expected, human CSF2 in these mice promoted the development of human alveolar macrophages in the lungs upon reconstitution with human immune cells (*SI Appendix*, Fig. S3 *C* and *D*).

#### Humanization of IL6.

Finally, we generated mice expressing human IL-6 by CRISPR-mediated gene replacement by inserting of coding regions of the human homolog (*SI Appendix*, Fig. S4*A*). Expression of IL-6 -was validated in an LPS induced inflammation model in blood (*SI Appendix*, Fig. S4*B*). As has been reported in our published work (31), humanization of IL-6 improved hematopoiesis upon engraftment of matched number of HSPCs (*SI Appendix*, Fig. S4*C*). Mice with human IL-6 had more human immune cells in blood at both 8 and 16 wk postengraftment than the corresponding strain lacking this cytokine gene (*SI Appendix*, Fig. S4*C*).

### Human Immune Cell Lineages in MaGIC and MISTRGv2 Mice.

We incorporated human CSF1(MCSF), CSF2 (GM-CSF), and IL-6 both in mice that harbor human CD47 (C^47^TRG) or human SIRPA (STRG), resulting in MaGIC and MISTRGv2 mice on the C57Bl/6 N background, making these mice highly relevant for mouse studies of the immune system as C57Bl/6 mice are the preferred choice in most mouse genetics and immunology research. These mice also encode human THPO and lack mouse *Rag1* and *Il2rg*. MaGIC and MISTRGv2 mice had similar levels of humanization, life span post reconstitution with human HSPCs and similar human lineages with a clear advantage in human neutrophil development in MaGIC mice (Fig. 1 *H*–*L*). A definitive identification of human neutrophils in MaGIC mice was achieved by Giemsa staining in blood smears (Fig. 1*M*). These cells had segmented nuclei with approximately three lobes, which is in accordance with the distinct morphology of mature human neutrophils (36). Overall, both MaGIC and MISTRGv2 mice had a similar life span of at least 18 wk upon engraftment and supported both lymphoid and myeloid lineages (Fig. 1 *H* and *J*). These mice also have mature human monocytes, tissue macrophages, alveolar macrophages, and dendritic cells provided by humanized M-CSF and GM-CSF and highly efficient hematopoiesis enhanced by IL-6 (*SI Appendix*, Figs. S2–S5), even when engrafted with adult BM derived human HSPC (Fig. 1 *K* and *L* and *SI Appendix*, Fig. S5 *A* and *B*).

Humanized MISTRG mice (transplanted with CD34^+^ cells) develop anemia with age (27, 29), which has been attributed to human myeloid cells particularly human macrophages failing to recognize mouse CD47, leading to the phagocytosis of mouse erythroid cells, including both precursors and mature red blood cells (RBCs). We hypothesized that expression of human CD47 would mitigate the loss of mouse RBCs and reduce anemia. To address this, we assessed RBC levels in vivo. Specifically, we compared RBC counts in engrafted and unengrafted MaGIC and MISTRGv2 mice (*SI Appendix*, Fig. S5*C*). In unengrafted mice, those expressing either human SIRPα or humanized CD47 exhibited similar RBC levels (*SI Appendix*, Fig. S5*C*). Unexpectedly, engrafted mice also showed comparable RBC levels and anemia, regardless of CD47 humanization (*SI Appendix*, Fig. S5*C*). Next, we assessed the ability of human macrophages to phagocytose mouse or human erythroid cells in vitro. Human macrophages were differentiated from peripheral blood monocytes and incubated with mouse RBCs expressing either mouse CD47 or human CD47 or with human RBCs. Phagocytosis in this set up showed little protective effect for mouse RBC expressing human CD47, supporting the in vivo results (*SI Appendix*, Fig. S5*D*). In contrast, human RBCs were not phagocytosed by human macrophages. Although we had hoped that human CD47 expression would protect mouse RBCs, these results suggest that any protective effect is insufficient to restore steady-state RBC levels or prevent anemia.

### Hematopoiesis in MaGIC Mice.

**Fig. 2.**
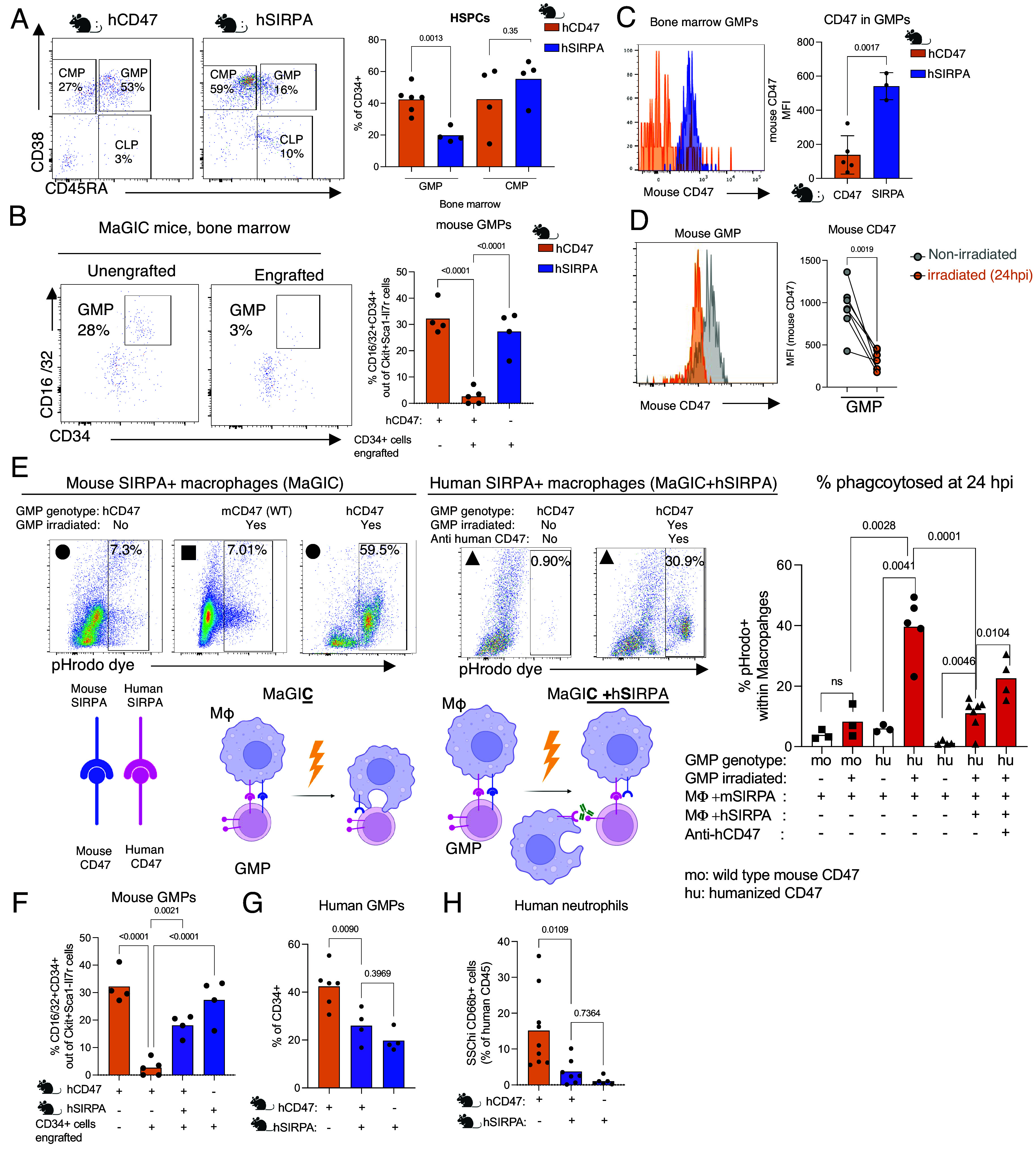
Human GMPs replace mouse GMPs that express low levels of mouse CD47 during emergency granulopoiesis. (*A*) Representative flow cytometry plots of human HSPCs highlighting cells enriched in human GMP expressing CD38 and high levels of CD45RA and cells enriched human CMP expressing CD38 and low levels of CD45RA from humanized mice with humanized CD47 or human SIRPA. Frequencies of HSPCs enriched in human GMPs and CMPs are plotted. Mean values are shown with data points. Statistical analysis was performed using the unpaired two-tailed *t* test. (*B*) Representative flow cytometry plots of mouse HSPCs marked by expression of CD16/32 and CD34 from humanized mice with humanized CD47 or human SIRPA that are engrafted or unengrafted. CD34 and CD16/32 expression is shown for mouse cells that are Lin−, Ckit+ Sca1−, IL7R− cells. Frequencies of mouse GMPs are plotted. Mean values are shown with data points. Statistical analysis was performed using the unpaired two-tailed *t* test. (*C*) Mouse CD47 expression on GMPs from humanized mice with humanized CD47 or human SIRPA. Mean values are shown with data points. Statistical analysis was performed using ordinary one-way ANOVA. (*D*) Mouse CD47 levels in irradiated or nonirradiated control GMPs that have been cultured for 24 h post irradiation (hpi). Mean values are shown with data points. Statistical analysis was performed using the paired two-tailed *t* test. (*E*) Proportions of macrophages that have phagocytosed mouse GMPs postirradiation. Mouse macrophages with and without human SIRPA were cultured with irradiated or nonirradiated sorted mouse GMPs that have humanized CD47 or wild type mouse CD47. Macrophage-GMP cultures were treated with anti-human CD47 blocking antibody when indicated. Mean values are shown with data points. Statistical analysis was performed using the unpaired two-tailed *t* test. (*F*) Frequencies of mouse GMPs in the BM of humanized mice with humanized CD47(MaGIC), humanized SIRPA(MISTRGv2), or both (MaGIC+hSIRPA) that are engrafted or unengrafted. Mean values are shown with data points. Statistical analysis was performed using ordinary one-way ANOVA. (*G*) Frequencies of human GMPs in the BM of humanized mice with humanized CD47(MaGIC), humanized SIRPA (MISTRGv2), or both (MISC^47^TRG6) that are immune reconstituted. Mean values are shown with data points. Statistical analysis was performed using ordinary one-way ANOVA. (*H*) Frequencies of human neutrophils in blood of engrafted humanized CD47(MaGIC), humanized SIRPA(MISTRGv2), or both (MaGIC+ hSIRPA). Mean values are shown with data points. Statistical analysis was performed using ordinary one-way ANOVA. All data are representative of at least two independent experiments.

Development of human neutrophils in MaGIC, but not MISTRGv2 mice prompted us to investigate the human HSPCs in BM of reconstituted mice. MaGIC BM had more HSPCs marked by total CD34 expressing cells within the nonlineage compartment of the BM (Fig. 2*A*). We further characterized the human HSPC subsets based on expression of CD38, CD45RA, and CD90. Interestingly, human HSPCs in MaGIC mice were preferentially enriched with granulocyte–macrophage progenitors (GMP) marked by high expression of both CD38 and CD45RA (Fig. 2*A*). There was a corresponding marked reduction in mouse GMP in these mice, enriched in progenitor cells expressing CD34 and CD16/32 (Fig. 2*B*). GMP, as implied in the name, give rise to neutrophils and monocytes, and their increase was in line with increased neutrophil and monocyte numbers in the circulation of mice with humanized CD47 (Fig. 1 *F* and *G*). CD47 expressed on the surface of all cells provides the “do not eat me” signal to its receptor SIRPA expressed on macrophages. Polymorphisms in *Sirpa* have been documented for *C57BL/6*, Balb/c, and NOD strains of mice. It has been shown that in protein binding assays, B6 SIRPA has a low affinity for human CD47 (37). This may suggest that certain murine cell types that heavily rely on CD47 are targeted for macrophage mediated removal and that niche availability changes in B6 humanized mice when mouse CD47 levels are particularly low (Figs. 1*B* and 2*C* and *SI Appendix*, Fig. S1*D*). In addition, loss of CD47 in the *C57BL/6* strain has been shown to favor xenotransplantation of human immune cells without need for human or polymorphic SIRPA (38). Based on these data we decided to test the role for SIRPA–CD47 interaction in the mechanism of CD47 mediated enhancement of certain HSPCs, particularly GMPs. We first confirmed that mouse CD47 expression on murine GMPs in MaGIC mice is indeed lower than in their counterpart MISTRGv2 mice in the *C57BL/6* background (Fig. 2*C*). Interestingly, loss of CD47 expression was more pronounced in GMPs compared with other mouse HSPCs such as closely related common myeloid progenitors (CMPs) (*SI Appendix*, Fig. S6*A*). Xenotransplantation in MaGIC mice requires conditioning with irradiation. We asked whether irradiation impacts CD47 levels in GMPs in MaGIC mice. Surprisingly, GMPs from MaGIC mice lost CD47 expression upon irradiation within 24 h (Fig. 2*D* and *SI Appendix*, Fig. S6*B*). GMPs irradiated with low and high doses of irradiation, exhibited reduced CD47 levels in a dose- and time-dependent manner (*SI Appendix*, Fig. S6 *B* and *C*). Loss of murine CD47 was again more pronounced in GMPs compared to CMPs at 24-h time point (*SI Appendix*, Fig. S6*D*). Based on these findings we hypothesized that loss of CD47 expression in GMPs upon irradiation rendered GMPs targets of mouse macrophages, opening the niche for human GMPs to take over myelopoiesis following irradiation. We tested this hypothesis utilizing in vitro phagocytosis assays that rely on pH-sensitive fluorogenic dyes which are nonfluorescent outside cells but fluoresce brightly in acidic pH environments, such as those of phagosomes, making them ideal for determining whether a given labeled cell type has been phagocytosed. Notably, irradiated GMPs, but not nonirradiated controls, were phagocytosed when cocultured with mouse macrophages, as indicated by the PhRodo fluorescent signal (Fig. 2*E*). The phagocytosis was found to be preventable if mouse macrophages expressed human SIRPA, facilitating interaction with human CD47 present on GMPs isolated from MaGIC mice. Consistent with this, when human CD47 was blocked using an antibody, the protective effect provided by SIRPA against phagocytosis was eliminated (Fig. 2*E*). Alternatively, in cultures where irradiated mouse LK cells were cocultured with mouse macrophages expressing mouse SIRPA, GMPs were preferentially lost (*SI Appendix*, Fig. S6*E*). However, GMPs were retained when they could interact with human SIRPA. (*SI Appendix*, Fig. S6*E*). To investigate whether a comparable effect occurred *in vivo*, we created MaGIC mice expressing human SIRPA (MaGIC + hSIRPA). As anticipated, the reconstituted MaGIC + hSIRPA mice exhibited elevated levels of mouse GMPs and reduced levels of human GMPs compared to MaGIC mice (Fig. 2 *F* and *G*). This shift in the human-to-mouse GMP ratio had consequences on circulating mature human neutrophil levels, resulting in a decrease (Fig. 2*H*).

Overall, these studies indicated that the loss of mouse CD47 expression postirradiation (in mice that already have reduced baseline mouse CD47 expression) created a BM environment that more effectively supports the progenitors of the myeloid lineage, resulting in enhanced development of human monocytes and neutrophils. In conclusion, human CD47 and SIRPA may differentially impact the BM niche that supports different progenitor cells and allow development of different immune cells. These effects may be further influenced by strain-specific variations in mouse SIRPα and its interaction with human CD47. To test strain specific effects in human immune cell phagocytosis, we have differentiated mouse macrophages from BALB/c and C57BL/6 mice. We chose BALB/c as our previous humanized mouse model was in the BALB/c background (26, 27, 29). We used pHrodo-labeled human cells to quantify phagocytosis by these mouse macrophages. As expected, the expression of human SIRPα in B6-derived mouse macrophages reduced the extent of human cell phagocytosis (*SI Appendix*, Fig. S6*F*). Next, we tested the effect of strain in the absence of human SIRPA. Interestingly, C57BL/6 mouse macrophages phagocytosed fewer human cells, suggesting that C57BL/6 SIRPα is more permissive in its interaction with human CD47 (*SI Appendix*, Fig. S6 *G* and *H*). This effect was most pronounced at 2 h of coculture but was no longer evident by 24 h (*SI Appendix*, Fig. S6*H*). Notably, macrophages from MaGIC mice consistently exhibited reduced phagocytosis of human immune cells at all time points (*SI Appendix*, Fig. S6*H*).

### Neutrophil Maturation Program and Subsets.

**Fig. 3.**
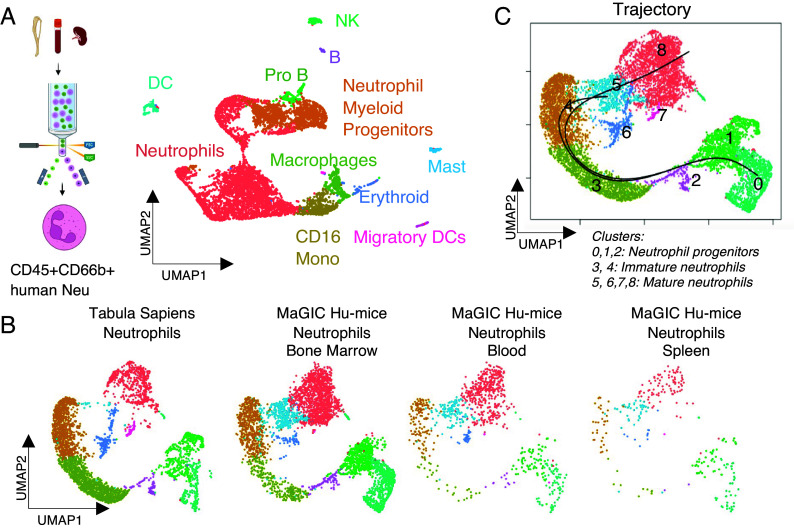
Transcriptional and developmental programs of human neutrophils from human donors (Tabula Sapiens) (40) and human neutrophils from Hu-mice (39) are equivalent. (*A*) UMAP visualization of sorted BM, blood, and spleen CD66b+ cells from reconstituted MaGIC mice analyzed by 10X Genomics Platform. Cells were labeled based on expression of known canonical markers genes and using an immune cell reference in from the package CellTypist (41, 42). (*B*) CCA-based integration of human neutrophils from Tabula Sapiens (40) and human neutrophils from Hu-mice separated by tissue (BM, blood, and spleen) (39). Canonical correlation analysis (CCA) was used to integrate and identify shared biological states across humanized and human data (43). CCA measures linear relationships between two sets of variables via correlation techniques to identify mutual nearest neighbors (MNNs) and transform the data across conditions while preserving sample-specific clusters postintegration (43, 44). (*C*) Gene trajectory analysis using Slingshot, mapping developmental trajectory of human neutrophils characterized as part of Tabula Sapiens from human donors and human neutrophils from Hu-mice.

Current studies also highlighted heterogeneity in neutrophils in circulation and tissues. In the context of cancer alone, there have been multiple neutrophil subsets with distinct molecular signatures in humans (21, 22). However, it is not clear whether these neutrophil types are truly distinct subsets or how they switch between healthy and diseases. To explore full neutrophil heterogeneity and differentiation landscape in a healthy state, we isolated human neutrophils and their progenitors from BM, blood, and spleen of reconstituted MaGIC mice. Transcriptome analysis of human neutrophils from MaGIC mice, referred to as human neutrophils from Hu-mice, at single cell level revealed multiple subsets and developmental stages of human neutrophils (39), including myeloid progenitors and mature human neutrophils (Fig. 3*A*). We then compared the transcriptome of human neutrophils from Hu-mice (39) to the transcriptome of human neutrophils described as part of Tabula Sapiens (40). Human and human neutrophils from MaGIC mice had completely overlapping clusters, recapitulating all developmental stages of neutrophils (Fig. 3*B* and *C* and *SI Appendix*, Fig. S7*A* and Dataset S1). We could map a developmental trajectory of neutrophils using Slingshot trajectory analysis and showed that while BM was enriched for both mature and immature neutrophils and progenitors, blood and spleens of MaGIC mice were primarily enriched for mature neutrophil subsets (Fig. 3 *B* and *C*). Mapping canonical markers of different stages of mouse neutrophil development (22) also confirmed the same trajectory in humanized mice (39) and humans (40) (*SI Appendix*, Fig. S7*B*) (22). Based on these studies (22), which established a developmental trajectory on a scale from G0 to G5, humanized BM neutrophils encompassed all these stages. In contrast, human neutrophils in the spleen and blood, similar to mouse neutrophils, were enriched for the G5 stage (*SI Appendix*, Fig. S7*B*). G0–G1 stages corresponding clusters 0 and 1 mainly include GMPs and committed neutrophil progenitors expressing *CD34, SOX4* and primary granule genes such as *MPO* (Fig. 3*C* and *SI Appendix*, Fig. S7*B*). G2–G3 stages are marked by expression of secondary granule genes such as *LTF* and *CAMP* (Fig. 3*C* and *SI Appendix*, Fig. S7*B*). G4 stage of human neutrophils from MaGIC mice marks the end of neutrophil maturation (Fig. 3*C* and *SI Appendix*, Fig. S7*B*). Clusters 3 and 4 represent G4 stage and are enriched for expression *MMP8*, a key granule enzyme for neutrophil-mediated host defenses, and *CXCR2* which is important for neutrophil mobilization. Cluster 5, 6, 7, 8 represent mature neutrophils enriched for genes that correspond to G5 stage, such as *FGL2*, *IFIT3*, *ISG15* (*SI Appendix*, Fig. S7*B*). G5 stage neutrophils make up the majority of blood and spleen neutrophils. Spleen neutrophils also exhibit tissue-specific adaptations, as demonstrated by their gene signature enriched for the IFNG response, in line with higher exposure to IFNG produced by T cells in the spleen (Dataset S2). Overall, these findings provide a comprehensive look at human granulopoiesis and human neutrophil development that is amenable to in vivo perturbations.

Finally, to determine neutrophil aging dynamics of human neutrophils, we used metabolic pulse and chase of neutrophils by labeling neutrophils with 5-Bromodeoxyuridine in vivo. This analysis at 2 and 5 d post injection demonstrated that the temporal changes in CD62L were different between mouse and human neutrophils (*SI Appendix*, Fig. S7*C*). BRDU+ mouse neutrophils showed reduced CD62L expression, consistent with earlier reports. In contrast, human neutrophils aged more slowly, as demonstrated by the persistent CD62L expression in BRDU+ human neutrophils, suggesting slowed neutrophil aging in humans (45).

### Human Neutrophils from Humanized Mice Are Functional.

**Fig. 4.**
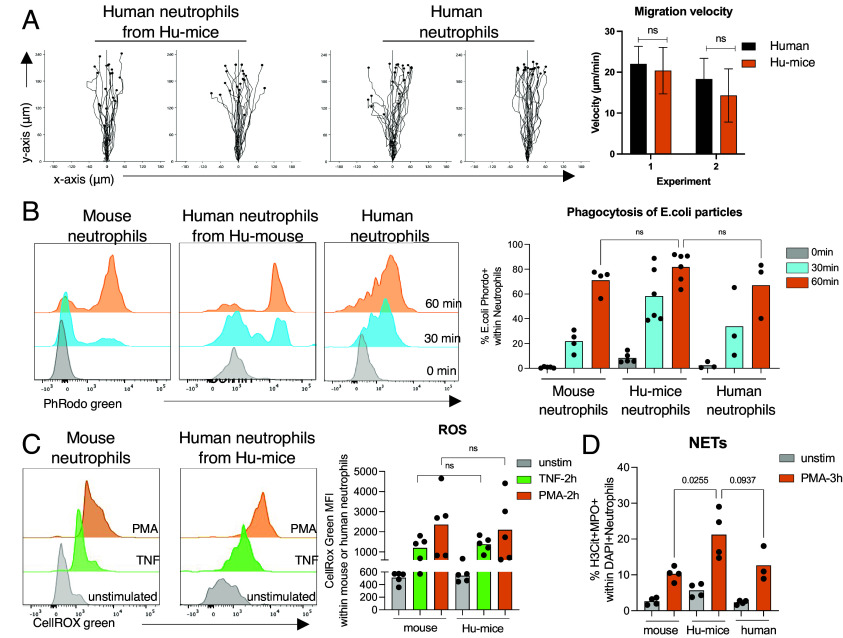
Human neutrophils in MaGIC mice are functional. (*A*) Ex vivo chemotactic ability of human neutrophils isolated from engrafted MaGIC mice and fresh human BM control were compared using the EZ-taxiscan chamber. Directionality and speed of human neutrophils were measured as they migrated toward IL8 (1 μM). Mean values are shown with data points. Statistical analysis was performed using the paired two-tailed *t* test. Image credit: Reprinted from ref. 26. (*B*) Representative flow cytometry plots of phagocytosis by human blood neutrophils (*Right*), human neutrophils from Hu-mice from the blood of engrafted MaGIC (*Middle*) and mouse neutrophils (*Left*). Blood samples were incubated with pHrodo Green *E. coli* BioParticles for the indicated times and fluorescent signals were analyzed by flow cytometry. *Lower* panel: 0 min. *Middle* panel: 30 min. *Top* panel: 60 min. Frequencies of pHrodo Green+ neutrophils are plotted. Mean values are shown with data points. Statistical analysis was performed using ordinary one-way ANOVA. (*C*) ROS production measured using CellRoxGreen assay in mouse or human neutrophils isolated from the BM of reconstituted MaGIC mice. Neutrophils were either stimulated with TNF of PMA for 2 h. Mean values are shown with data points. Statistical analysis was performed using the paired two-tailed *t* test. (*D*) Frequencies of mouse humanized and human neutrophils undergoing NETosis upon PMA stimulation for 3 h. NETosis was measured by flow cytometry based on costaining of citrullinated Histone 3 (H3Cit) and MPO within DAPI+ neutrophils. Mean values are shown with data points. Statistical analysis was performed using ordinary one-way ANOVA. All data are representative of at least two independent experiments.

Neutrophils are indispensable during injury, infection, and, as more recently appreciated, in homeostatic immune surveillance in both humans and mice (3–6). They play essential roles in defense against microbial infections through a variety of mechanisms including chemotaxis to sites of inflammation, phagocytosis, release of ROS, and by their unique formation of NETs (6, 8) We tested whether human neutrophils from Hu-mice could perform these essential effector functions characteristic of neutrophil biology. First, to test the chemotactic ability of these human neutrophils from Hu-mice we compared the directionality and speed of human neutrophils as they migrated toward IL-8 ex vivo using the EZ-TAXIS can chamber. The human neutrophils from humanized mice were indeed capable of chemotaxis (Fig. 4*A* and Movies S1 and S2). Both the directionality and migration speed of the human neutrophils from Hu-mice were comparable to those of neutrophils from healthy human donors. Next, we tested the functional capacity of reconstituted human neutrophils to phagocytose inflammatory targets in an in vitro assay using fluorogenic *Escherichia coli* bioparticles (46). Human neutrophils from MaGIC blood were capable of phagocytosis with efficacy similar to human neutrophils from human blood or to mouse neutrophils (Fig. 4*B*). Another essential feature of neutrophils is their ability to produce ROS upon inflammatory stimulation. We quantified ROS levels in humanized and mouse neutrophils upon inflammatory TNF stimulation or PMA treatment. Human neutrophils from Hu-mice responded to both stimuli and produced high levels of ROS measured by a fluorogenic probe detecting oxidative stress in live cells (Fig. 4*C*). Formation of NETs is another unique feature of neutrophils. Human neutrophils from Hu-mice, like their mouse and human counterparts, effectively formed NETs upon stimulation measured by flow cytometric evaluation of MPO and citrullinated histone H3 (Fig. 4*D*). Overall, these findings indicate that human neutrophils from Hu-mice are fully functional and respond appropriately to inflammatory stimuli, similar to their human and mouse counterparts.

## Discussion

Neutrophils play a vital role in defending against injury and infection by engaging in phagocytosis, releasing ROS, antimicrobial peptides, proteases, and forming NETs. While these functions are conserved, significant species-specific differences can be observed in the maturation, migration, and function of neutrophils. Humanized mice are invaluable for studying human immune development and function in vivo, but absence of mature human neutrophils in circulation and tissues has been a major limitation. To overcome this, we generated a version of the humanized mouse model named MaGIC in the C57Bl/6 N strain that improves human myelopoiesis and enables development of functional human neutrophils. Consequently, these mice exhibit 5 to 35% human mature neutrophils in peripheral blood, compared with ~1% in other humanized mouse models. Mechanistically, the mouse GMP niche became available to human GMPs and particularly myelopoiesis as a result of the irradiation-induced complete loss of CD47 in mouse GMPs and their elimination by mouse macrophages. In addition to human neutrophils, MaGIC mice also have mature human monocytes, tissue macrophages, alveolar macrophages, and dendritic cells supported by humanized M-CSF and GM-CSF (25). All human neutrophil subsets described in human BM and blood are present and phenotypically equivalent to primary human cells in MaGIC mice. These human neutrophils are functional and perform essential neutrophil functions in vitro and in vivo. Therefore, MaGIC mice have significant implications for understanding various pathologies, including hyperinflammatory responses in chronic viral infections and autoimmune and inflammatory diseases, paving the way for more faithful translational research that focus on human neutrophils, the most abundant human immune cell.

The preferential reduction of mouse CD47 levels on GMPs could be explained by variable levels of CD47 protein on different immune cells and their progenitors. Interestingly, it has been reported that GMPs, especially when mobilized, have higher expression of CD47 compared with other HPSCs (47). It is possible that reduced or lost murine CD47 expression on BM resident GMPs preferentially tags these murine progenitor cells for removal, opening niche availability in the BM for human GMPs. Although MaGIC mice do not express human SIRPa, modification of CD47 allows engraftment of human cells in the mouse host. Our results also argue that there may be nonoverlapping complementary functions of CD47 and SIRPA that should be further investigated either individually or in combination.

The effectiveness of the model hinges on the reduction of CD47 levels following irradiation. However, this process can be adversely impacted by various factors that elevate CD47 expression, including inflammation prior to engraftment, highlighting the need for enhanced husbandry practices. It is also unclear whether other chemical or genetic conditioning approaches, such as busulfan or mutations in the c-Kit gene (48), could be used to enhance BM niche availability.

In humanized mice, certain mouse cells—such as RBCs—are targeted and eliminated by developing human macrophages. We hypothesized that expressing human CD47 on these mouse cells could prevent their phagocytosis by human macrophages which express human SIRPA. This rationale guided our CD47 gene expression design, in which all cells that normally express mouse CD47 also express human CD47 in the appropriate location, timing, and amount. While CD47 humanization may have some effect, it was not sufficient to fully prevent the anemia observed at steady state in mice bearing high levels of human tissue macrophages, which are primarily supported by human M-CSF. We have previously shown that humanization of the mouse liver in cytokine humanized mice prevents phagocytosis of human red cells by eliminating mouse complement C3 (49). Additional factors such as cell rigidity, shape, and opsonization (as seen for human RBC in humanized mice) could play a role in phagocytosis, overriding signals provided by CD47 (49, 50).

At steady state levels of human neutrophils among human immune cells in mouse blood remains below frequencies observed in human blood. However, inflammatory infectious stimuli increase these frequencies markedly increased, suggesting a role for persistent inflammatory stimuli in maintenance of high neutrophil proportions possibly due to induction of human cytokines such as G-CSF that play a role in human neutrophils’ maintenance and mobilization. In our earlier work, we have shown that targeting the G-CSF–G-CSFR axis improves circulation of human neutrophils in MISTRG6 (version 1) mice in the Balb/c background (26). We have observed that mouse G-CSF works equally well as human G-CSF on human neutrophils in vitro using a cell survival assay as the readout (*SI Appendix*, Fig. S8 *A* and *B*). It is possible that the effects mediated through the G-CSF–G-CSFR axis in human neutrophil development in humanized mice are largely due to the impaired responsiveness of mouse neutrophils, which lack the G-CSF receptor, rather than the humanization of G-CSF. It remains to be studied whether humanization of G-CSF or deletion of mouse G-CSFR will have a similar, additional impact in MaGIC mice.

Targeting the G-CSF receptor axis partially improves human neutrophil levels but requires depleting mouse neutrophils, increasing susceptibility to infections and complicating breeding and inflammatory disease studies. In contrast, mice with humanized CD47 show normal neutrophil development and maintenance at steady state without irradiation, resulting in reduced infection susceptibility, and improved breeding efficiency and lifespan, as confirmed by intact GMP frequencies and neutrophil function assay in unirradiated MaGIC mice. It was essential to maintain low levels of mouse CD47 expression at steady state without irradiation, as mice completely lacking mouse CD47 (we regenerated mouse *Cd47* knockout mice on the *Rag1^−/−^* and *IL2rg^−/−^* background) exhibited very poor health and breeding performance.

The MaGIC mouse model improved human myelopoiesis through a mechanism involving reduced CD47 in mouse progenitors, thereby enhancing niche availability for human cells and enabling robust human neutrophil development and function. This model offers significant potential for studying neutrophil dynamics in multiple human diseases such as infections and inflammatory diseases and cancer, providing an incisive in vivo platform for therapeutic interventions.

## Materials and Methods

### Mice.

The generation of MaGIC and MISTRGv2 was performed on a *C57Bl/6 N* background with CRISPR guides designed to target regions of mouse *Rag1, Il2rg, Cd47, Sirpa, Csf1, Csf2, Il6* genes. Visual representation of gene constructs, and targeting are provided as part of main and supplementary figures and also discussed in *SI Appendix*, Table S1.

#### Mouse Rag1 deletion.

CRISPR guide targets in the N terminus of the coding exon of *Rag1* gene and downstream of the stop codon in the 3′ UTR mediate the deletion of a ~3.2 kb genomic segment (*SI Appendix*, Fig. S1*A*).

#### Mouse Il2rg deletion.

CRISPR guide targets in the regions of the *Il2rg* gene upstream of the transcription start site and downstream of the polyadenylation site were employed to excise a ~4.3 kb genomic segment encompassing the entire gene (*SI Appendix*, Fig. S1*B*).

#### Humanization of mouse Thpo gene.

Using CRISPR-mediated, homology-directed integration, a human *THPO* complete coding (cDNA) sequence (red rectangle) of ~1 kb has been inserted into the 5′ UTR of the mouse Thpo gene (*SI Appendix*, Fig. S1*C*). The primary goal was to insert a human coding region at the translational start of the mouse Thpo gene so that expression of the human protein would be placed under the control of the 5′ regulatory architecture of the mouse gene. This approach involved inserting the human coding sequence into exon 2, thus preserving the 5′ UTRs split between exons 1 and 2 and the intervening first intron that displayed sequence conservation between mouse and human and that also contained a potential regulatory region as identified by REMAP ChIP-seq data. Since the 3′ mouse UTR displayed human-mouse sequence conservation and a REMAP peak, we also wanted to retain that portion of the transcriptional unit. Finally, since other introns in the mouse gene also displayed human-mouse sequence conservation and a REMAP peak was present in intron 4, the entire gene structure surrounding the human coding region insertion was retained. Although there was a potential risk of nonsense-mediated mRNA decay, we confirmed THPO protein expression and found it sufficient to support human platelet development, indicating functional activity.

#### CD47 humanization.

An IRES (black rectangle, Fig. 1*A*) and a human CD47 complete coding (cDNA) sequence (adjoining red rectangle, Fig. 1*A*) of ~1 kb have been inserted immediately downstream of stop codon in the last exon of the mouse Cd47 gene via CRISPR-mediated, homology-directed integration. This design preserves both upstream and intronic regulatory elements governing expression of the mouse gene. Human SIRPA protein levels and reduced mouse CD47 levels were quantified by antibody staining (Biolegend) followed by analysis via flow cytometry.

#### Humanization of the mouse Sirpa gene.

The replacement of key genomic sequences was carried out by homologous recombination in C57BL/6 ES cells. Exons 2 to 4 of the mouse *Sirpa* gene (Fig. 1*A*, top) have been replaced with the corresponding exons (coding sequences in red) from the human SIRPA gene (Fig. 1*A*, bottom). A 7.9 kb segment has been integrated into the mouse gene so that the encoded chimeric protein has the mouse signal sequence (mouse exon 1) followed by the entire human extracellular region corresponding to human amino acids 28 to 362 (human exons 2 to 4) fused to the intracellular portion of the mouse SIRPα protein (mouse exons 5 to 8) for proper signaling in mouse cells. Human SIRPA protein expression was quantified by antibody staining followed by analysis via flow cytometry.

#### Mouse Csf1 gene humanization.

Using CRISPR-mediated, homology-directed integration, exon 2 of the mouse *Csf1* gene has been replaced with a human *CSF1* cDNA (in red as shown in *SI Appendix*, Fig. S2*A*), starting with amino acid 14 of the signal sequence (which is homologous in mice and humans) and containing the full remainder of the human coding region (1,626 bp). A truncated human 3′ UTR (in blue as shown in *SI Appendix*, Fig. S2*A*) preserves several identified functional elements responsible for regulating *CSF1* mRNA turnover (35), including a common microRNA target region, a G-quadruplex noncanonical tetrahelix and AU-rich elements (AREs), and this is then followed by a strong transcriptional terminator from SV40 (in black, as shown in *SI Appendix*, Fig. S2*A*). In this design, the mouse promoter, exon 1, and intron 1 are left intact, so that splicing reconstitutes the complete coding sequence. Transcription termination immediately following the human coding region is designed to prevent transcription of the downstream portion of the mouse *CSF1* gene and thus eliminate nonsense-mediated decay of the humanized mRNA. The complete mouse/human hybrid coding region was sequence-confirmed. Human M-CSF protein expression was quantified by ELISA (R&D Systems).

#### Mouse Csf2 gene humanization.

Excision of the mouse *Csf2* gene (~2 kb, top line) and concomitant insertion of the similarly sized human *CSF2* homolog (in red as shown in *SI Appendix*, Fig. S3*A*) has been engineered via a CRISPR-mediated, targeted integration, effectively placing the human transcriptional unit under the control of the mouse promoter and upstream regulatory elements. Mouse 3′ UTR sequences that govern mRNA stability have been preserved in this design. Expression of the human coding sequence mRNA and protein has been confirmed. Human GM-CSF protein expression was quantified by ELISA (R&D Systems).

#### Mouse Il6 gene replacement and validation.

*IL6* construct Excision of the mouse segments (~6.3 kb, top line as shown in *SI Appendix*, Fig. S4*A*) and insertion of the coding regions of the human IL6 homolog (~4.3 kb, bottom line, exons and introns in red as shown in *SI Appendix*, Fig. S4*A*) has been engineered employing a homology-dependent repair strategy via CRISPR. Expression of the human coding sequence mRNA and protein has been confirmed. Human IL6 protein levels in serum were quantified by ELISA (R&D Systems).

The original MISTRG6 mouse in the Balb/c background used in some of the phagocytosis assays has been generated in collaboration with Regeneron Pharmaceuticals and published earlier (29, 33). All animal experimentations were performed in compliance with Yale Institutional Animal Care and Use Committee protocols. MaGIC mice will be made available from Yale under a material transfer agreement. Instructions on obtaining the material transfer agreement for this mouse strain will be available along with strain information upon request.

### Human Immune Cell Reconstitution and CD34+ HSPC Injection.

Prior to human CD34+ HSPCs injection, mice were first conditioned by irradiation (X-ray irradiation with X-RAD 320 irradiator). The irradiation dose, ranging from 250 cGy to 320 cGy, was empirically optimized to facilitate human immune cell engraftment while maximizing survival. In the MaGIC mouse that incorporates all necessary factors for human hematopoiesis, an irradiation dose of 250 cGy was used. Adult (6-wk old) mice weighing at least 18 g were irradiated the day before transplantation with human HSPC. Unless otherwise specified, mice were retro-orbitally injected into the venous sinus with 100 μL of PBS containing either 100,000 FL– or cord blood–derived CD34^+^ cells, or 200,000 BM CD34^+^ cells, using a 28-gauge needle. All use of human materials were deemed exempt from human subject research and was approved by the Yale University Human Investigation Committee. All human materials were deidentified prior to use in your study.

### Characterization of Human Immune Cells by Flow Cytometry.

All mice were analyzed at approximately 6 to 18 wk postengraftment as described before (26). Blood was collected retro-orbitally in EDTA coated or heparinized collection tubes. Samples were stained by an antibody cocktail at room temperature for 20 min and then treated with RBC lysis/fixation solution (Biolegend #422401). Fixed cells were then washed and resuspended with PBS for analysis. BMs (femurs) were dissected and flushed with PBS supplemented with 2% FBS and 1 mM EDTA to form single-cell suspensions, which were subsequently treated with ammonium-chloride-potassium (ACK) lysis buffer to eliminate RBCs as described before (26). Lung tissues were first chopped into small pieces, digested in RPMI1640 medium supplemented with collagenase D (1 mg/mL) for 35 min at 37 °C, and then filtered through 70 μm cell strainers (Fisher Scientific #07-201-431) to form single cell suspensions (51). Single-cell suspensions isolated from BM, lung, blood, and spleen were stained with antibody cocktail on ice for 15 min and then washed with FACS buffer and fixed with 1% Formalin or 2% paraformaldehyde for 30 min. Fixed cells were then washed and resuspended in PBS for analysis.

### Human Neutrophil Isolation.

Human neutrophils were isolated from blood by density gradient centrifugation (Lymphoprep Density Gradient Medium, Stemcell), resuspended in PBS with 2% FBS and 1 mM EDTA. The polymorphonuclear and erythrocyte later was treated with equal volume of ACK lysing buffer, centrifuged (1,500 rpm, 10 min), and resuspended in HBSS.

### Cell Sorting.

For cell sorting experiments of neutrophils, single-cell suspensions from BM, spleen, and blood of reconstituted MaGIC mice were stained with antibodies against human CD45, mouse CD45, mouse CD66b and sorted using the BigFoot Spectral Cell Sorter (Thermo Fisher Scientific). Cell viability was assessed prior to sort with DAPI prior to sort and with AOPI after the sort.

For cell sorting experiments of GMPs and LK cells, BM cells were isolated from femurs of MaGIC (humanized CD47) or MITRGv2 mice (wild type mouse CD47). Single-cell suspensions of BM cells were stained with antibodies against mouse lineage (Ly6c, Ly6G, CD11b, Ter119), mouse CD34, mouse C-kit, mouse Sca1, mouse CD16/32.

### In Vitro Differentiation of BM Derived Macrophages.

Under sterile conditions, BM cells were isolated from femurs of MISTRG6v2 mice, MaGIC mice or MaGIC mice encoding human SIRPA. For differentiation into human macrophages in vitro, BM cells were incubated in medium supplemented with 10% FBS, 1% penicillin–streptomycin and recombinant mouse M-CSF (50 ng mL^−1^) at 1 × 10^6^ per mL concentration for 6 d in an incubator under 5% CO_2_ and at 37 °C. Medium supplemented with 10% FBS was replenished with new medium every 3 to 4 d. Bone-marrow-derived macrophages were monitored for granularity, elongated morphology, and stronger adherence to the plate. These macrophages were used in phagocytosis assays.

### Chemotaxis.

Human neutrophils were isolated by positive selection using EasySep protocols according to the manufacturer’s instructions and as described before (26). The EZ-TAXIScan (ECI Frontier, MIC-1000) was used to investigate chemotaxis of human neutrophils as described before (26, 52). After removal of bubbles and heating of the chamber to 37 °C, 10,000 neutrophils in 1.5 μL of HBSS supplemented with 0.1% endotoxin-free BSA were loaded into the lower reservoir. 1 μL of 1 μM IL8 was loaded into the upper reservoir. Time lapse images were taken for 20 min at 30-s intervals. At least 20 cell tracks from each sample were analyzed using Gradientech Tracking Tool™ PRO v2.1 (free software). Chemotaxis speed and directionality toward IL8 were determined by the cell tracks. Upward directionality was calculated based on the center of mass and accumulated distance.

### In Vitro Phagocytosis Assays.

#### Functional characterization of neutrophils.

Isolated human neutrophils from cord blood were incubated with pHrodo Green *E. coli* BioParticles (Life Technologies; 1 mg/mL) at 37 °C for 30 min (10 µg *E. coli* particles per 100 µL cells) as described before (26). These particles coated with pH sensitive dyes fluoresce when phagocytosed and are in the acidic environment of endosomes or lysosomes. Cells were fixed with 1% formalin and fluorescence of pHrodo conjugates was analyzed using flow cytometry.

#### Phagocytosis of labeled mouse cells by macrophages.

FACS sorted mouse irradiated (200 to 250 cGy) or nonirradiated GMPs and labeled with Sartorius pHrodo Cell Labeling kit following the manufacturer’s instructions. Briefly, cells were washed with prewarmed pHrodo wash buffer. Cells were then resuspended in pHrodo labeling buffer at a 1 × 10^6^ cell per ml. Cell Labeling Dye for Incucyte is dissolved in DMSO at 1 mg/mL concentration and diluted as necessary. GMPs were incubated at 0.5 μg/mL concentration of dye for 30 min at 37 °C. Cells are centrifuged at 1,000 rpm for 10 min and washed with RPMI complemented with 10% FBS and 1% penicillin–streptomycin (the same media used for macrophages differentiation). Once washed, labeled GMPs were added on cultures of BM-derived macrophages for 24 h.

#### Phagocytosis of labeled mouse RBCs by human macrophages.

RBCs (1 × 10^9^ cells) from mice encoding human SIRPA or human CD47 or RBCs from human cord blood were washed and labeled with pHrodo dye as described before (53). These labeled RBC were then cocultured with human macrophages for an in vitro erythrophagocytosis assay. To each well seeded with human macrophages, roughly 4.5 million labeled RBCs were added. Human macrophages were differentiated from human blood monocytes with human MCSF as described before. The cocultures were then incubated for 2 h at 37 °C in a humidified incubator with 5% CO_2_. Human macrophages were then fixed with 1% Formalin and analyzed by flow cytometry.

### ROS Production Assay.

BM human neutrophils and mouse neutrophils were isolated as described for previous assays following the manufacturer’s instructions (CellROX® Oxidative Stress Reagents, ThermoFisher) (26). Briefly, isolated BM neutrophils were stimulated with PMA (50 ng/mL) for or with human or mouse TNFa (20 ng/mL) for 2 h at 37 °C 30 min. In the last 30 min of 2-h incubation, CellROX reagents at a final concentration of 5 μM were added to the cells. Cells were washed, stained with anti-human CD66b APC antibody and fixed. ROS production was analyzed by flow cytometry using mean fluorescent intensity as a readout.

### NETosis Assay.

Isolated human neutrophils were cultured with 100 nM Phorbol-1-myristate-13-acetate (PMA) for 3 h at 37 °C as described before (26). Following stimulation, cells were treated with ACK lysing buffer and fixed in 2% PFA for 30 min at RT. Fixed cells were washed and then stained with DAPI and the following antibodies in PBS: APC/Cy7 anti-mouse Ly-6G antibody (BioLegend, Clone: 1A8, 1:100 dilution), APC anti-human CD66b antibody (BioLegend, Clone: G10F5), FITC anti-human myeloperoxidase (MPO) antibody (Life Technologies, Clone: MPO455-8E6), and anti-human Histone H3 (citrulline R2,R8,R17, Abcam RM1001) antibody conjugated to Cyanine 647 using Mix-n-Stain™ Antibody Labeling Kits (Biotium). Data were acquired with a LSRII flow cytometer (BD Biosciences) and analyzed using FlowJo v10.10. Human NET-forming neutrophils are DAPI^+^ CD66b^+^ MPO^+^ H3Cit^+^.

### Single Cell RNA-Sequencing Analysis.

Neutrophils from the BM, spleen, and blood of humanized MaGIC mice were sorted based on human CD66b (a surface marker for human neutrophils) and human CD45 expression. Sorted cells from blood and spleen were labeled with barcoded hashing antibodies (TotalSeqB0251, TotalSeqB0252; BioLegend) and pooled with cells from BM and loaded onto the 10× Chromium Controller using the Chromium Next GEM technology. Library preparation was performed in-house using the Chromium Next GEM Single Cell 3′ Reagent Kits v3.1 (10× Genomics), including cell surface feature barcoding. Sequencing was conducted on the NovaSeq 6000 system (Illumina).Raw sequencing data were processed using Cell Ranger (v7.1.0) and aligned to the GRCh38-2020-A reference genome. The filtered feature-barcode matrix from Cell Ranger was analyzed in R (v4.2.3) using Seurat (v5.0.1). Low-quality cells (percent.mt < 12%, nCountRNA < 500, nFeatureRNA < 125) were excluded. Data were normalized and scaled (ScaleData). Cells were demultiplexed using hashing barcodes: TotalSeqB0251 and TotalSeqB0252 identified blood and spleen cells, while unassigned cells were labeled as BM. Cell clusters were annotated using canonical markers and refined with CellTypist (41). Although neutrophils were significantly enriched, other cell types were also recovered due to less stringent sorting aimed at maximizing cell viability. All downstream analysis was performed on the neutrophil subsets. These human neutrophils derived from MaGIC (39) were then integrated with human neutrophils from the Tabula Sapiens (40) to compare those from humanized mice with those from human donors. To infer potential stages of neutrophil maturity throughout the blood, BM, and spleen, we used Slingshot (54) for trajectory inference and highlighted genes that are representative of various stages of neutrophil maturity (22).

### Statistical Analysis.

Student’s *T* test (paired or unpaired and two-tailed) or one-way ANOVA was used to determine statistical significance. One-way ANOVA was employed for comparing multiple data groups against controls. For single cell analysis, *P*-values were calculated using the moderated *t* test (Limma R package) or the Wilcoxon method. For multiple testing correction, the false discovery rate was calculated, and Benjamini–Hochberg correction was applied.

## Supplementary Material

Appendix 01 (PDF)

Dataset S01 (CSV)

Dataset S02 (CSV)

Movie S1. Human neutrophils from bone marrow of reconstituted MaGIC mice chemotaxing towards IL8. The EZ-TAXIScan (ECI Frontier, MIC-1000) was used to investigate chemotaxis of human neutrophils. Related to figure 4A. Movies are generated using Image J.

Movie S2. Human neutrophils from fresh human bone marrow chemotaxing towards IL8. The EZ-TAXIScan (ECI Frontier, MIC-1000) was used to investigate chemotaxis of human neutrophils. Related to figure 4A. Movies are generated using Image J. This human reference data, analyzed side-by-side with human neutrophils from MaGIC mice, was previously published in Zheng and Sefik et al (2).

## Data Availability

Raw scRNA sequencing data, processed count matrices, and an R object with neutrophils from scRNA sequencing data generated in this study were deposited to the NCBI Gene Expression Omnibus under the Accession No. GSE303007 (39). MaGIC mice are available from Yale under a material transfer agreement. Previously published data were used for this work [https://cellxgene.cziscience.com/e/4f1555bc-4664-46c3-a606-78d34dd10d92.cxg/ (40); (26)]. See *SI Appendix*, *SI Materials and Methods* for more detailed description of experimental methods and analysis. All other data are included in the manuscript and/or supporting information.
