# Supplementary material for: Humanization of CD47 enables development of functional human neutrophils via postirradiation remodeling of the bone marrow
Source: Proc Natl Acad Sci U S A. 2025 Sep 16;122(38):e2426546122. doi: 10.1073/pnas.2426546122 (PMC12478129; doi:10.1073/pnas.2426546122)
Supplement: Supplementary file 1 — Appendix 01 (PDF) [file pnas.2426546122.sapp.pdf]

PNAS

Supporting Information for
Humanization of CD47 enables development of functional human neutrophils via postirradiation remodeling of the bone marrow.

Esen Sefik[a,1,2], esen.sefik@yale.edu, William Philbrick[b,1], Fengrui Zhang[a], Kriti Agrawal[a,c], Brian Van Lee[a], Johannes Sam[d], Kutay Karatepe[e], Yunjiang Zheng[a], Kaixin Liang[a], Sophia Peng[a], Haris Mirza[f], Athreya Rangavajhula[a], Perrine Simon[a], Neha Arun[g], Priyanka Babu[a], Elizabeth Eynon[a], Michael Chiorazzi[a], Liang Shan[h], Stephanie Halene[f,i], Hongbo R. Luo[j], Anthony Rongvaux[k], Yuval Kluger[c,f,l], and Richard A. Flavell[a,m,2], richard.flavell@yale.edu

Author Affiliations
[a] Department of Immunobiology, Yale School of Medicine, New Haven, CT 06519
[b] Department of Internal Medicine, Section of Endocrinology, Yale School of Medicine, New Haven, CT 06519
[c] Computational Biology and Biomedical Informatics Program, Yale University, New Haven, CT 06519
[d] Roche Innovation Center Zurich, Schlieren 8952, Switzerland
[e] Department of Cell Biology, Yale School of Medicine, New Haven, CT 06519
[f] Department of Pathology, Yale School of Medicine, New Haven, CT 06519
[g] Department of Microbial Pathogenesis, Yale School of Medicine, New Haven, CT 06519
[h] Division of Infectious Diseases, Department of Medicine, Washington University School of Medicine in St. Louis, St. Louis, MO 63110
[i] Department of Internal Medicine, Yale Comprehensive Cancer Center, Yale University School of Medicine
[j] Department of Pathology, Brigham and Women's Hospital, Boston, MA 02115
[k] Translational Science and Therapeutics Division, Fred Hutchinson Cancer Center, Seattle, WA 98109
[l] Program in Applied Mathematics, Yale University, New Haven, CT
[m] HHMI, Yale University School of Medicine, New Haven, CT
[2] To whom correspondence may be addressed. Email: esen.sefik@yale.edu or richard.flavell@yale.edu.
Contributed by Richard A. Flavell; received December 18, 2024; accepted August 7, 2025; reviewed by H. L. Grimes and Markus G. Manz
[1] E.S. and W.P. contributed equally to this work.

This PDF file includes:

SI Materials and Methods
Figures S1 to S8
Tables S1
Legends for supplementary movies S1 to S2
Legends for Datasets S1 to S2
SI References

Other supporting materials for this manuscript include the following:

Supplementary Movies 1 and 2
Datasets S1 to S2

Supporting Information Text
SI Materials and Methods

Human CD34$^+$ hematopoietic stem and progenitor cell isolation

Human CD34+ hematopoietic stem and progenitor cells were isolated and injected into recipient mice as previously described(1).

Fetal liver: De-identified human fetal tissues were obtained from Cercle Allocation Services (CA) and Advance Bioscience Resources Inc (CA), not-for-profit organizations, established exclusively for medical, scientific and educational purposes. Briefly, human fetal liver samples were cut in small fragments, treated for 20-45 min at 37 °C with collagenase D (Roche, 1mg/ml), and prepared into a cell suspension as described before(2). Human CD34+ cells were purified from single cell suspensions by performing density gradient centrifugation (Lymphocyte Separation Medium, Sigma-Aldrich/PromoCell), followed by positive immunomagnetic selection with EasySep™ Human CD34 Positive Selection Kit (Stemcell Technologies). The purity of the positively selected CD34+ was confirmed by flow cytometry (over 95%). CD34$^+$ cells were frozen in FBS containing 10% DMSO and stored long-term in liquid nitrogen.

Cord blood: Cord blood was acquired from Yale Allocation Services. Human CD34$^+$ HSPCs were isolated from cord blood via density gradient centrifugation (Lymphoprep Density Gradient Medium, Stemcell), resuspended in PBS with 2% FBS and 1 mM EDTA, and subjected to positive immunomagnetic selection using EasySep Human CD34 Positive Selection Kit II (Stemcell). CD34$^+$ and CD34$^-$ fractions were counted and checked for purity by staining with PE anti-human CD34 antibody (Biolegend #, Clone 581:). CD34$^+$ cells were frozen in FBS containing 10% DMSO and stored long-term in liquid nitrogen.

Bone marrow: Frozen human CD34$^+$ stem cells derived from bone marrow were acquired from Ossium Inventa and stored long-term in liquid nitrogen.

For engraftment, frozen CD34+ cells were thawed and recounted in PBS medium. and resuspended in PBS in appropriate volume for injection. Cells were pre-warmed prior to injection.

Characterization of human immune cells by flow cytometry

Single cells suspensions isolated from bone marrow, lung, blood and spleen were stained with antibody cocktail on ice for 15 min and then washed with FACS buffer and fixed with 1% Formalin or 2% paraformaldehyde for 30 min as described before(3). Fixed cells were then washed and resuspend in PBS for analysis. Antibodies against the following antigens were used at final concentration of 0.5 μg/ml: Mouse antigens: FITC anti-mouse CD45 antibody (Biolegend, Clone: 30-F11), Brilliant violet 605 anti-mouse CD45 antibody (Clone: 30-F11), APC/Cy7 anti-mouse Ly-6G antibody (Biolegend, Clone: 1A8), Alexa Fluor 488 anti-mouse Ly-6C antibody (Biolegend, Clone: HK1.4). Human antigens: Brilliant violet 605 anti-human CD45 antibody (Clone: HI30), APC/Cy7 anti-human CD3 antibody (Biolegend, Clone: HIT3a), PerCP/Cyanine 5.5 anti-human CD3 antibody (Biolegend, Clone: HIT3a), APC/Cyanine 7 anti-human CD10 antibody (Biolegend, Clone: HI10a), PerCP/Cyanine 5.5 anti-human CD16 antibody (Biolegend, Clone: 3G8), APC/Cyanine 7 anti-human CD14 antibody (Biolegend, Clone: HCD14), Brilliant Violet anti-human CD68 antibody (Biolegend, Clone: Y1/82A), PE/Cy7 anti-human CD19 antibody (Biolegend, Clone: HIB19), APC anti-human CD33 antibody (Biolegend, Clone: WM53), APC/Cyanine 7 or PE anti-human CD34 antibody (Biolegend, Clone: 561), PE or APC anti-human CD66b antibody (Biolegend, Clone: G10F5), PE/Cy7 anti-human CD101 (BB27) antibody (Biolegend, Clone: BB27), Pacific Blue or PE Cy7 anti-human CD335 (NKp46) antibody (Biolegend, Clone: 9E2). Data were acquired with FACSDiva 7 on an LSRII flow cytometer (BD Biosciences) and analyzed with FlowJo v10 software.

LPS stimulation

Mice were injected with LPS (Enzo Life Sciences) at a dose of 1mg/kg intraperitoneally as described before(2, 4). Blood and tissues were collected 2 hours post injection. For human IL6 protein quantification, serum was used; for human GM-CSF quantification, lung homogenates that were lysed in RIPA buffer were used.

Conjugation of Antibodies

Anti-Histone H3 (citrulline R2 + R8 + R17) antibody from Abcam (RM1001) was conjugated using Biotium Mix-n-Stain CF Dye Antibody Labeling Kit following manufacturer's instructions. After completing the pre-labeling check list, 100 micrograms of total antibody was diluted in Mix-n-Stain Reaction Buffer. The entire solution was then added to dye/label in the provided vial. The vial was incubated in the dark for 15 minutes at room temperature. After incubation, 300 microliters of storage buffer were added. The labeled antibody was transferred to light protective tubes and stored at 4C.

Single Cell RNA-sequencing analysis

Neutrophils from the bone marrow, spleen, and blood of humanized MaGIC mice were sorted based on human CD66b (a surface marker for human neutrophils) and human CD45 expression. Sorted cells from blood and spleen were labeled with barcoded hashing antibodies (TotalSeqB0251, TotalSeqB0252; BioLegend) and pooled with cells from bone marrow to a final concentration of 1,500 cells/µL and loaded onto the 10x Chromium Controller using the Chromium Next GEM technology. Library preparation was performed in-house using the Chromium Next GEM Single Cell 3' Reagent Kits v3.1 (10x Genomics), including cell surface feature barcoding. Sequencing was conducted on the NovaSeq 6000 system (Illumina). Raw sequencing data were processed using Cell Ranger (v7.1.0) and aligned to the GRCh38-2020-A reference genome. The filtered feature-barcode matrix output from Cell Ranger was used for downstream analysis in R (v4.2.3) with the Seurat package (v5.0.1). Low quality cells, defined as those with high mitochondrial transcript contents (percent.mt < 12%) and low RNA counts (nCountRNA < 500, nFeatureRNA < 125), were excluded from the dataset and the subsequent analysis pipeline included normalization, data scaling using ScaleData, and principal component analysis. Cells were demultiplexed based on the hashing antibody barcodes. Cells with TotalSeqB0251 and TotalSeqB0252 barcodes were assigned as blood and spleen cells respectively, and unassigned cells were labeled as bone marrow cells. Cell clusters were annotated based on the expression of known canonical marker genes and refined using CellTypist with the immune cell reference model (Immune_All_Lowpkl). Cells were then filtered based on their mitochondrial reads and RNA counts. Cells were annotated based on expression of known canonical markers genes and using an immune cell reference in from the package CellTypist (5, 6). Although neutrophils were significantly enriched, other cell types, including monocytes, macrophages, and B cells, were also recovered. This was due to less stringent sorting conditions designed to maximize viability and prioritize speed during cell sorting. Since we were specifically interested in the neutrophils, we subsetted neutrophils and performed all downstream analysis on these cells. These neutrophils were then integrated with human neutrophils from the Tabula Sapiens allowing comparison of those derived from MaGIC mice with those from human donors. To infer potential stages of neutrophil maturity throughout the blood, bone marrow, and spleen, we used Slingshot (7) for trajectory inference and highlighted genes that are representative of various stages of neutrophil maturity (8).

Integration with Tabula Sapiens Bone Marrow data

Human Bone Marrow single-cell data were downloaded from the Tabula Sapiens website (https://cellxgene.cziscience.com/e/4f1555bc-4664-46c3-a606-78d34dd10d92.cxg/) and integrated with our pre-processed humanized single-cell data using Canonical Correlation Analysis (CCA) to correct for batch effects. Neutrophils were selected based on CSF3R positivity from the

dataset and clustered at a resolution of 0.3, followed by UMAP embedding. Marker genes for each cluster were identified using "FindAllMarkers()", and a heatmap was generated based on the top 5 marker genes for each cluster (shown in Fig. S7A).

RNA Trajectory analysis

Slingshot(7) was employed to perform trajectory analysis on single-cell RNA-seq data, aiming to infer cellular transitions between clusters. Cells were grouped into distinct clusters based on their gene expression profiles, which were subsequently visualized using UMAP. The black lines overlaying the plot (starts from cluster 0) represent trajectories connecting these clusters, illustrating the inferred lineage progression and continuous cell state transitions.

Visualization of Neutrophils differentiation and maturation

Differentiating and mature neutrophils were visualized transcriptionally using feature plots based on established gene signatures(8). The early stages of neutrophil maturation were represented by genes such as CD34, SOX4, MPO, LTF, and CAMP, while the later stages were characterized by genes including MMP8, CXCR2, ISG15, IFIT3, and FGL2.

G-CSF–mediated in vitro survival assay of human neutrophils

Human neutrophils were isolated as previously described using a density gradient and resuspended at 5×10^6 cells per mL in RPMI supplemented with 10% FBS. Cells were cultured in vitro for 48 hours in the presence of recombinant human or mouse granulocyte colony-stimulating factor (G-CSF) at a final concentration of 10 ng/mL, as described before (mouse GCSF purchased from TargetMol, human GCSF from Proteintech) (9). Following incubation, cell viability and metabolic activity were quantified using the CellTiter-Glo Luminescent Cell Viability Assay (Promega), following the manufacturer's instructions.

Supplementary Figures.

Fig. S1: Humanization of CD47 yields more myeloid cells in tissues.

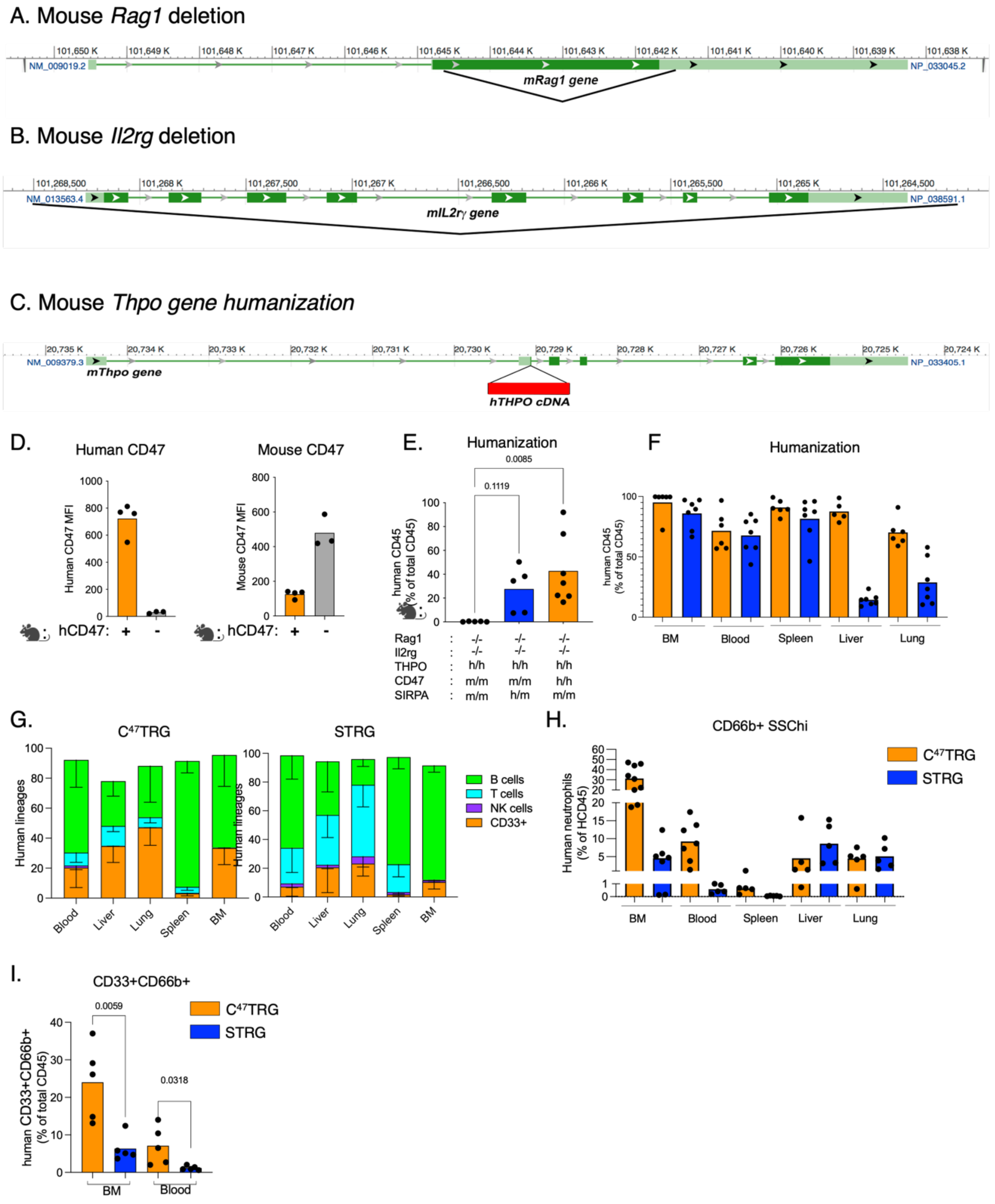

Fig S1: Humanization of CD47 yields more myeloid cells in tissues.

A. Mouse *Rag1* deletion. CRISPR guide targets in the N-terminus of the coding exon and downstream of the stop codon in the 3' UTR mediated the deletion of a ~3.2 kb genomic segment. Dark green denotes coding segments, and light green denotes non-coding segments.

B. Mouse *Il2rg* deletion. CRISPR guide targets in the regions of the gene upstream of the transcription start site and downstream of the polyadenylation site were employed to excise a ~4.3 kb genomic segment encompassing the entire gene.

C. Humanization of *THPO*: Using CRISPR-mediated, homology-directed integration, a human *THPO* complete coding (cDNA) sequence (red rectangle) of ~1 kb has been inserted into the 5' UTR of the mouse *Thpo* gene. The goal was to insert the human coding region at the translational start of the mouse *Thpo* gene to place its expression under mouse regulatory control while preserving conserved UTR, introns, and REMAP-predicted regulatory elements. Although this design risked nonsense-mediated decay, we proceeded to generate the model and empirically assessed expression levels and functionality.

D. Mean fluorescent intensity of mouse and human CD47 expression in mice with or without humanized CD47.

E. Humanization in immunodeficient mice expressing human SIRPA, human CD47, or neither (control). Mice were irradiated with a dose of approximately 320 cGy and engrafted with 200,000 human CD34+ HSPCs. Humanization was measured as the proportion of human immune cells out of mouse and human cells in blood of reconstituted mice. Mean values are shown with data points. Statistical analysis was performed using ordinary one-way ANOVA.

F. Humanization measured as frequencies of human immune cells within total immune cells (mouse and human) in mice with humanized SIRPA or CD47.

G. Human immune cell lineages in blood, liver, lung, spleen and bone marrow (BM) of humanized CD47 or SIRPA.

H. Frequencies of human neutrophils in blood, liver, lung, spleen and bone marrow (BM) of humanized CD47 or SIRPA encoding mice, reconstituted with human CD34+ HSPCs. Mean values with standard deviation (SD) are shown.

I. Frequencies of human CD33+CD66b+ immune cells in bone marrows (BM) and blood of humanized CD47 or SIRPA. Mean values are shown with data points. Statistical analysis was performed using unpaired two-tailed t-test.

Fig S2: Humanization of CFS1/M-CSF supports all subsets of human monocytes. human tissue macrophages and human NK cells

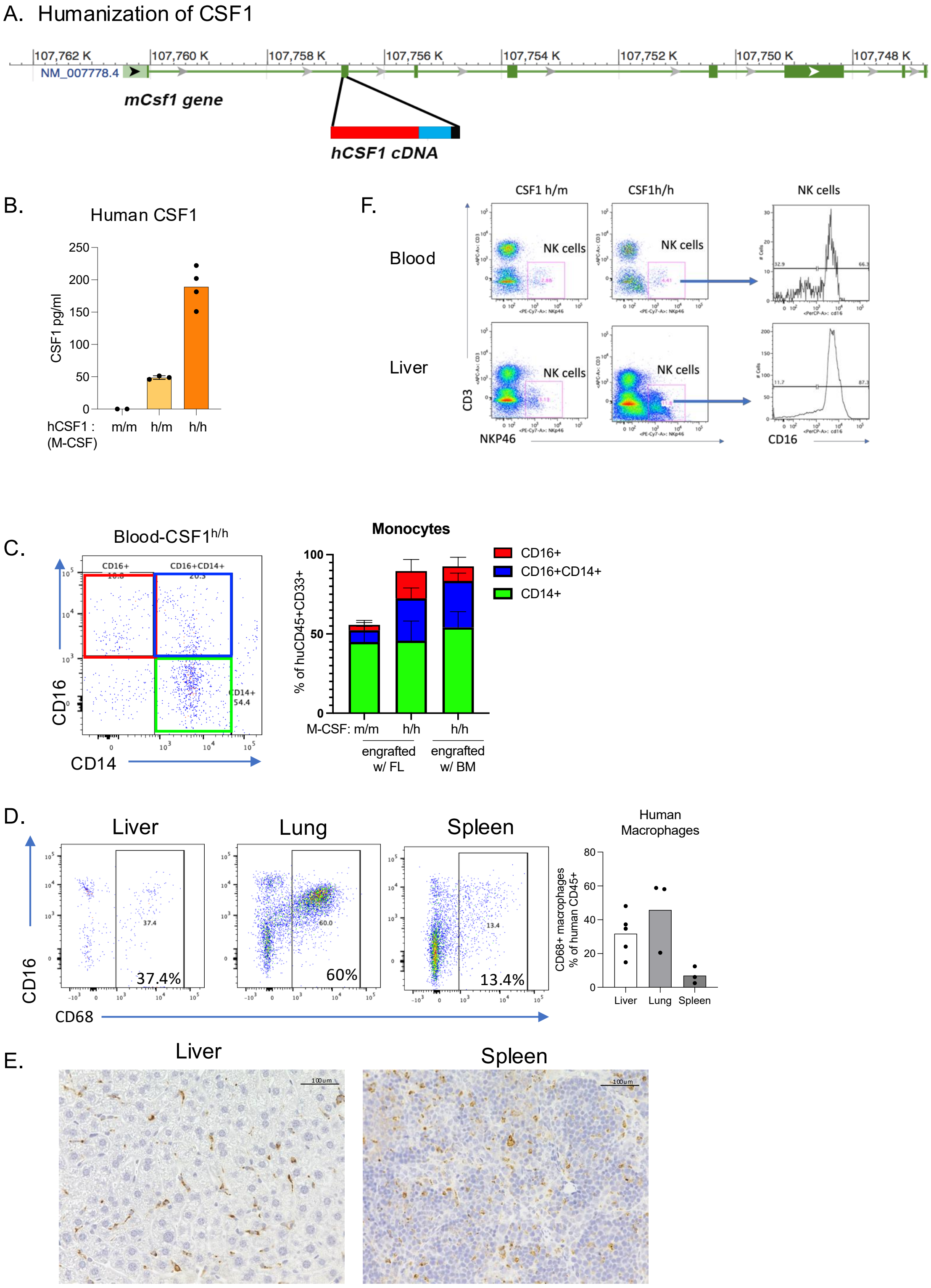

Fig. S2: Humanization of CSF1/M-CSF supports all subsets of human monocytes. human tissue macrophages and human NK cells

A. Mouse *Csf1* gene humanization and validation: Using CRISPR-mediated, homology-directed integration, exon 2 of the mouse *Csf1* gene has been replaced with a human *CSF1* cDNA (in red), starting with amino acid 14 of the signal sequence (which is homologous in mouse and human) and containing the full remainder of the human coding region (1626 bp). A truncated human 3' UTR (in blue) preserves several identified functional elements responsible for regulating *CSF1* mRNA turnover(10), including a common microRNA target region, a G-quadruplex noncanonical tetrahelix and AU-rich elements (AREs), and this is then followed by a strong transcriptional terminator from SV40 (in black). In this design, the mouse promoter, exon 1 and intron 1 are left intact, so that splicing reconstitutes the complete coding sequence. Transcription termination immediately following the human coding region is designed to prevent transcription of the downstream portion of the mouse *Csf1* gene and thus eliminate nonsense-mediated decay of the humanized mRNA. The complete mouse/human hybrid coding region was sequence-confirmed and the expression of the humanized mRNA and protein has been confirmed by RTq-PCR and ELISA, respectively.

B. Quantification of serum human CSF1 protein levels in C57BL/6 (B6) control, *CSF1* h/h (encoding homozygous human CSF1) and *CSF1* h/m (encoding heterozygous human CSF1) mice and in humans.

C. Representative flow cytometry plot and frequencies of all subsets (CD14+, CD14+CD16+, CD16+) of human monocytes in humanized mice encoding human CSF1 or not. Mice were engrafted with CD34+ cells isolated from fetal liver (FL) or bone marrow (BM). Mean values with SD are shown.

D. Representative flow cytometry plots and frequencies of human tissue macrophages (CD68+) in liver, lungs and spleen of mice with humanized CSF1.

E. Immunohistochemistry images of human CD68 staining of liver and lungs of immune reconstituted humanized mice encoding human *CSF1*. Representative of n=4 mice.

F. Representative flow cytometry plots and frequencies of all subsets of circulating and tissue resident human NK cells in liver, lung, and spleen of mice with humanized CSF1.

Fig. S3: : Humanization of CSF2/GM-CSF provides human alveolar macrophages.

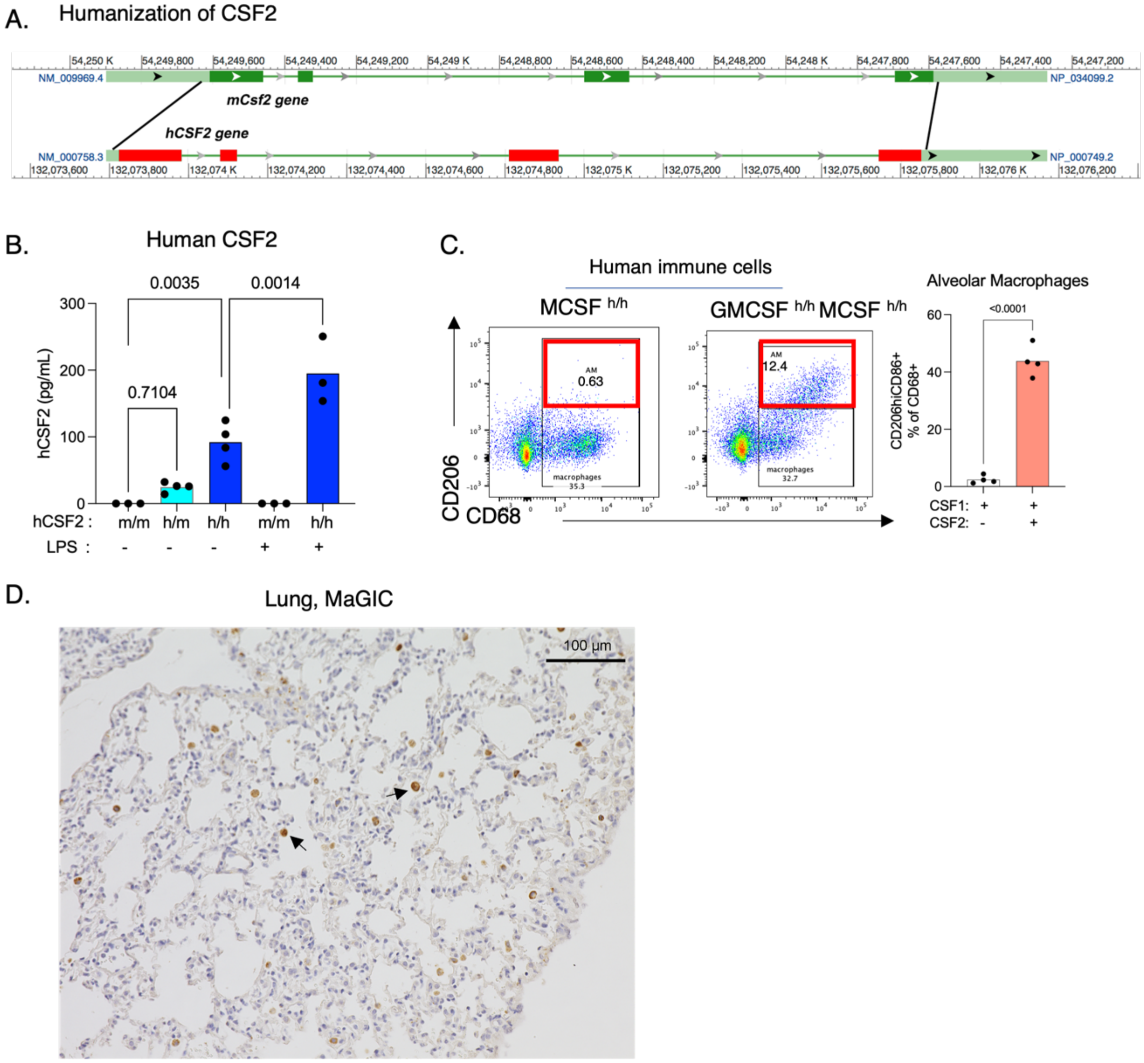

Fig. S3: : Humanization of *CSF2* provides human alveolar macrophages.
A. Mouse *Csf2* gene humanization and validation: Excision of the mouse gene (~2 kb, top line) and concomitant insertion of the similarly sized human *CSF2* homolog (in red) has been engineered via a CRISPR-mediated, targeted integration, effectively placing the human transcriptional unit under the control of the mouse promoter and upstream regulatory elements. Mouse 3' UTR sequences that govern mRNA stability have been preserved in this design. Expression of the human coding sequence mRNA and protein has been confirmed by RTq-PCR and ELISA, respectively.
B. Human CSF2 protein levels in lungs of mice encoding mouse *CSF2* (m/m) or human *CSF2* (h/m and h/h) at steady state or upon LPS treatment. Mean values are shown with data points. Statistical analysis was performed using unpaired two-tailed t-test.
C. Representative flow cytometry plots and graphical representation of frequencies of alveolar macrophages marked by co-expression of high levels of CD206 and CD68 in mice that encode human *CSF2* and *CSF1* or just human *CSF1*. Mice expressing human CSF1 develop some lung tissue macrophages but lack human alveolar macrophages. Mean values are shown with data points. Statistical analysis was performed using unpaired two-tailed t-test.
D. Immunohistochemistry images of CD68 staining lungs of immune reconstituted humanized mice encoding human *CSF1* and human *CSF2*. Black arrows point to alveolar macrophages. Representative of n=4 mice.

Fig. S4. Humanization of cytokine IL-6 improves human hematopoiesis.

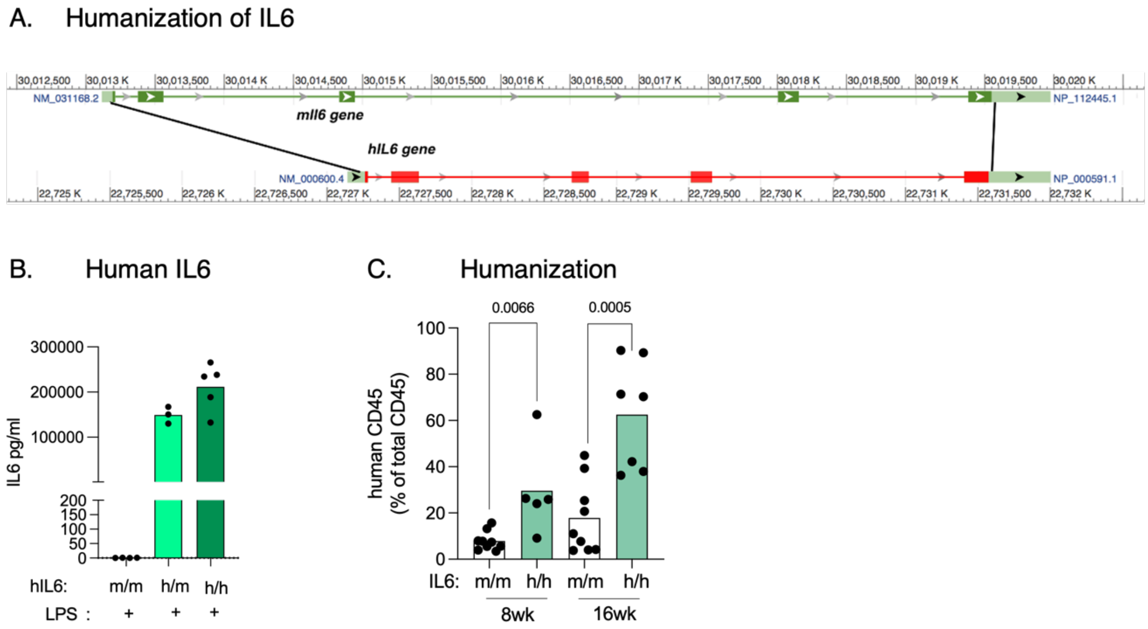

Fig. S4. Humanization of cytokine IL-6 improves human hematopoiesis.

A. Mouse *Il6* gene replacement and validation: Excision of the mouse segments (~6.3 kb, top line) and insertion of the coding regions of the human IL6 homolog (~4.3 kb, bottom line, exons and introns in red) has been engineered employing a homology-dependent repair strategy via CRISPR. Expression of human IL6 has been confirmed by qPCR and ELISA

B. Human IL6 protein levels in serum of B6 *IL6* m/m (control), *IL6* h/m and IL6 h/h mice upon LPS treatment. Mice were injected intraperitoneally with LPS (1mg/kg). Serum was collected 2 hours post injection. Mean values are shown with data points. Statistical analysis was performed using unpaired two-tailed t-test.

C. Humanization measured as frequencies of human cells (human CD45+) among all immune cells (human or mouse CD45+) in humanized mice with and without human IL6. Mean values are shown with data points. Statistical analysis was performed using paired two-tailed t-test.

Fig. S5. Humanization of CD47 is not sufficient to prevent anemia.

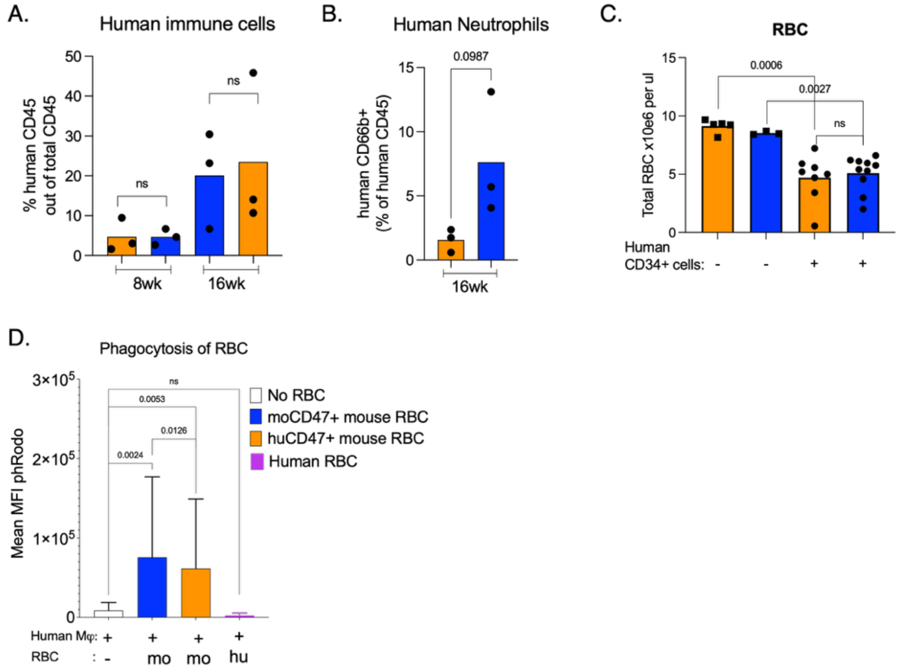

Fig. S5. Humanization of CD47 is not sufficient to prevent anemia.

A. Humanization as measured by frequencies of human cells (human CD45+) among all immune cells (human or mouse CD45+) in blood of humanized mice encoding human SIRPA (MISTRG) or human CD47 (MaGIC). Mice were engrafted with bone marrow derived human CD34+ HSPC. Mean values are shown with data points. Statistical analysis was performed using unpaired, two-tailed t-test.

B. Frequencies of human neutrophils in blood of humanized mice encoding human SIRPA (MISTRG) or human CD47 (MaGIC). Mice were engrafted with bone marrow derived human CD34+ HSPC. Mean values are shown with data points. Statistical analysis was performed using unpaired, two-tailed t-test.

C. RBC levels in engrafted (circles) and unengrafted (squares) age- matched humanized mice that express human SIRPA (MSTRG6 mice) or humanized CD47 (MaGIC mice). Mean values are shown with data points. Statistical analysis was performed using unpaired, two-tailed t-test.

D. In vitro phagocytosis of mouse RBC by human macrophages. Labeled mouse red blood cells (RBCs) (with pHrodo dye as described before (11)) were co-cultured with human macrophages for an in vitro erythrophagocytosis assay. Co-cultures were then incubated for 2 hours. Repeated at least 3 independent times with 3 donors. Mean values with standard deviation are shown. Statistical analysis was performed using ratio paired, two-tailed t-test.

Fig. S6. Loss of mouse CD47 on GMPs upon irradiation make them susceptible to phagocytosis.

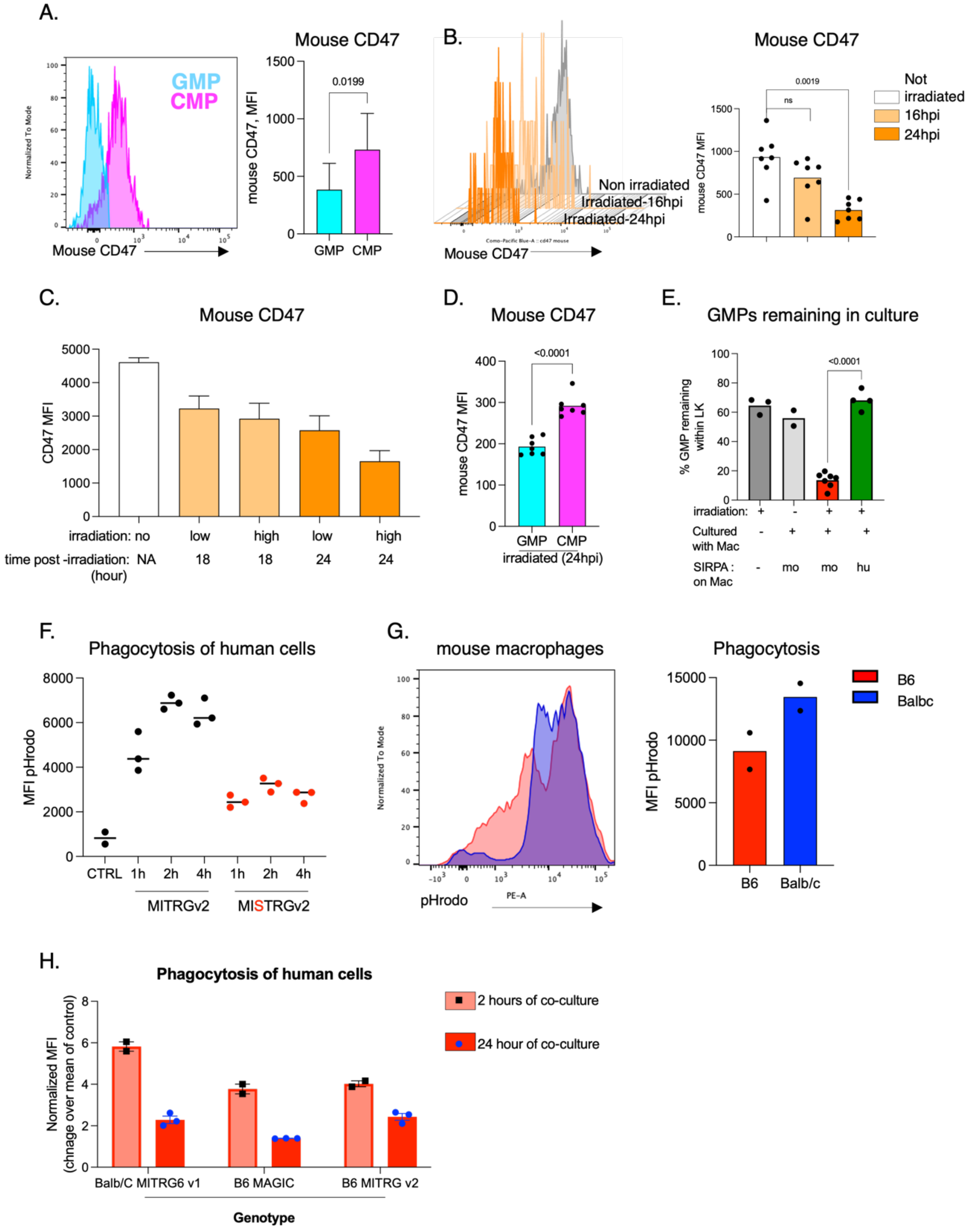

Fig. S6. Loss of mouse CD47 on GMPs upon irradiation make them susceptible to phagocytosis.

A. Mouse CD47 expression, quantified as mean fluorescent intensity (MFI), on GMPs and CMPs from reconstituted humanized mice with humanized CD47. Mean values with SD are shown. Statistical analysis was performed using paired, two-tailed t-test.

B. Representative histograms and graphical representation of MFI of mouse CD47 levels on GMPs that were irradiated with a high dose irradiation for either 16 hours or 24 hours or left unirradiated.

C. Mouse CD47 levels (measured as MFI) on mouse GMP that were irradiated with a high (250cGy) or low dose (150 cGy) irradiation for either 16 hours or 24 hours or left unirradiated.

D. Mouse CD47 expression, quantified as MFI, on GMPs and CMPs upon irradiation (24 hours post irradiation-hpi). Bone marrow cells were enriched for mouse LK cells based lineage identifying markers, C-kit and Sca-1 expression. Lin- Ckit+ Sca1- cells were irradiated, and bone marrow cells were cultured for 24 hours prior to staining with a cocktail of antibodies for identification of mouse GMPs and CMPs and mouse CD47. Mean values are shown with data points. Statistical analysis was performed using paired, two-tailed t-test.

E. Frequencies of GMP remaining in the supernatants of LK-macrophage cultures. Irradiated or non-irradiated LK cells from MaGIC mice were cultured with mouse macrophages expressing human or mouse SIRPA. Mean values are shown with data points. Statistical analysis was performed using unpaired, two-tailed t-test.

F. Effects of human SIRPA on phagocytosis of human cells by mouse bone marrow derived macrophages from C57BL/6 (B6) MITRGv2 and MISTRGv2 mice.

G. Representative histograms of pHrodo intensity and graphical representation of mean florescent intensity (MFI) of pHrodo as a measure of phagocytosis of human cells. Mouse macrophages differentiated from bone marrows of B6 or BALB/c mice (MITRG) were co-cultured with human cord blood mononuclear cells.

H. Normalized MFI (change over mean of control macrophages) as a measure of phagocytosis of human cord blood cells by mouse macrophages from MITRG6-BALB/c (described before(12)), MITRG6 v2-B6 and MaGIC- B6 mice. Two time points are presented (2 and 24 hours of co-culture).

Fig S7. Transcriptional, developmental programs and aging dynamics of human and human neutrophils from Hu-mice are equivalent.

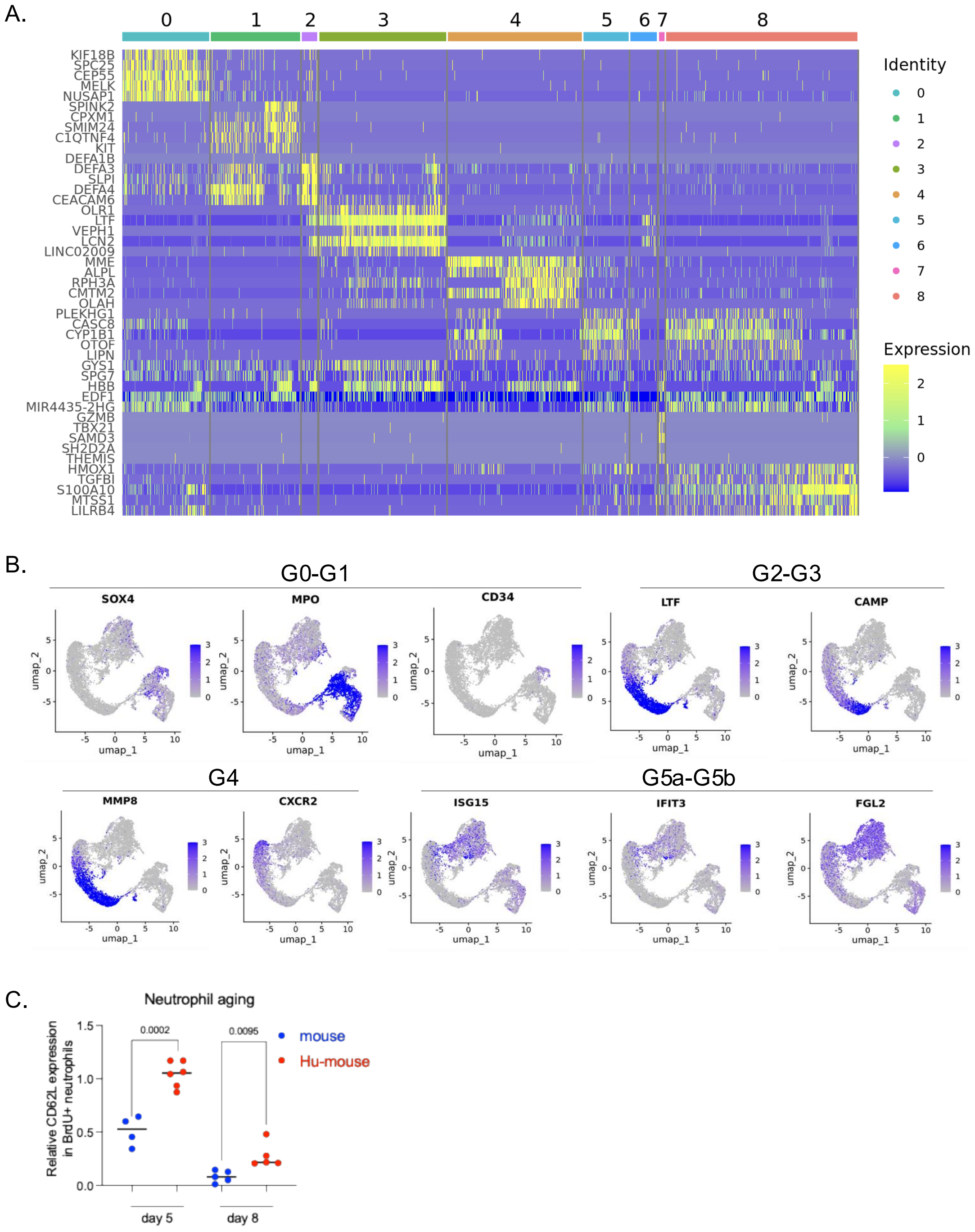

Fig S7. Transcriptional, developmental programs and aging dynamics of human and human neutrophils from Hu-mice are equivalent.

A. Heatmap of cluster identifying genes post-integration for the 9 clusters identified as part of Fig.3B-C.

B. UMAP visualization of genes that mark different developmental stages of neutrophils(8). G0-G1 stages refer to neutrophil and myeloid progenitors; G2-G4 stages refer to immature neutrophils. G5a,b stages refer to mature neutrophils.

C. Neutrophil aging comparing human neutrophils from humanized mice and mouse neutrophils. Temporal changes in CD62L expression were measured following metabolic pulse and chase of neutrophils with bromodeoxyuridine (BrdU) in vivo. This assay that relies on CD62L, a marker reduced during aging as described in previous studies to demonstrate neutrophil aging (13). Mean values are shown with data points. Statistical analysis was performed using unpaired two-tailed t-test.

Fig. S8 Mouse and human G-CSF are cross reactive in vitro.

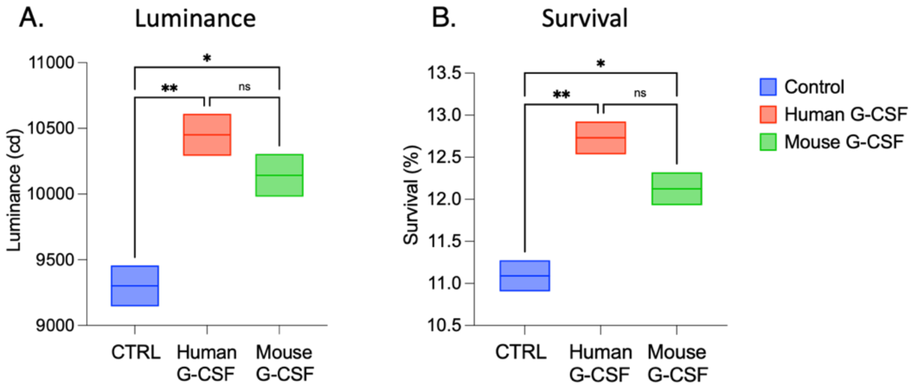

Fig. S8 Mouse and human G-CSF are cross reactive in vitro.

A. CellTiter-Glo Luminance and **B.** survival of human neutrophils cultured for 48 hours in presence of human or mouse granulocyte colony-stimulating factor (G-CSF) or in absence of any GSCF. Human neutrophils from blood were cultured with human or mouse G-CSF at a concentration of 10 ng/mL, using a cell density of 5 million neutrophils per mL. After incubating for 48 hours, cellular responses using the CellTiter-Glo assay were measured as the readout.

Supplementary Tables

Table S1: Approach, function, and limitations of genes that have been modified in humanized mice.

Gene	Function / immune cell	Genetic approach	Limitation
CSF1 (MCSF)	Myeloid cells (and lymphocytes): Human CSF1 enables development of all monocyte subsets and tissue macrophages. Human NK cells are supported indirectly by human IL15 produced by these myeloid cells.	Exon 2 of the mouse *Csf1* gene has been replaced with a human *CSF1* cDNA, starting with amino acid 14 of the signal sequence. A truncated human 3' UTR preserves several identified functional elements responsible for regulating CSF1 mRNA turnover. The mouse promoter, exon 1 and intron 1 are left intact, so that splicing reconstitutes the complete coding sequence. Construct generated by CRISPR-mediated homology directed repair.	Human macrophages phagocytose mouse red blood cells, leading to anemia.
CSF2 (GMCSF)	Myeloid cells: enables development of alveolar macrophages and improves dendritic cell responses.	Excision of the mouse *Csf2* gene (exons and introns, ~2 kb) and concomitant insertion of the similarly sized human *CSF2* homolog. The human transcriptional unit is under the control of the mouse promoter and upstream regulatory elements, including mouse 3' UTR sequences that govern mRNA stability. Construct generated by CRISPR.	Mice develop alveolar proteinosis due to elimination of mouse alveolar macrophages. Requires enhanced husbandry practices.
IL6	Hematopoiesis and immune response: Increased hematopoiesis efficiency. Improved B cell response and enhanced immature myeloid cells.	The genomic segment encompassing the entire coding region of the mouse *Il6* gene (exons and introns, ~6.3 kb) has been replaced with the homologous coding regions of the human *IL6* gene (~4.3 kb) using a homology-directed repair strategy via CRISPR.	
CD47	Xeno-tolerance, improved human myelopoiesis, and functional human neutrophil development. This is achieved by reduced expression of murine CD47 in HSPCs, particularly GMPs.	An internal ribosomal entry site (IRES) and a human CD47 complete coding (cDNA) sequence (~1 kb) have been inserted immediately downstream of stop codon in the last exon of the mouse *Cd47* gene. This design preserves both upstream and intronic regulatory elements governing expression of the mouse gene. This approach allowed human *CD47* to be expressed at physiological levels but resulted in significantly reduced levels of mouse CD47.	Requires irradiation for engraftment. Factors that elevate CD47 levels may adversely impact humanization.
SIRPA	Xeno-tolerance, human hematopoiesis. Humanization of SIRPA prevents phagocytosis of human cells by mouse macrophages, enabling human hematopoiesis.	Exons 2-4 of the mouse *Sirpa* gene have been replaced with the corresponding exons from the human SIRPA gene by homologous recombination in C57BL/6 ES cells. A 7.9 kb segment has been integrated into the mouse gene so that the encoded chimeric protein has the mouse signal sequence (mouse exon 1) followed by the entire human extracellular region corresponding to human amino acids 28 to 362 (human exons 2 to 4) fused to the intracellular portion of the mouse SIRPα protein (mouse exons 5 to 8) for proper signaling in mouse cells.	
THPO	Hematopoiesis: maintenance of HSC, lifelong hematopoiesis, platelets. Human THPO supports human platelet development.	A complete human THPO coding (cDNA) sequence inserted into the 5' UTR of the mouse *Thpo* gene so that expression of the human protein is under the control of the 5' regulatory architecture of the mouse gene. This strategy inserts the human coding sequence into exon 2 while retaining conserved mouse regulatory elements—including the 5′ UTRs (exons 1–2 and intron 1), the 3′ UTR, and all flanking introns with identified potential regulatory elements—to preserve endogenous transcriptional control.	Additional defects in human platelet development and maintenance, similar to human erythropoiesis.
Rag1	Deletion of mouse *Rag1* gene eliminates T and B lymphocytes.	CRISPR guide targets in the N-terminus of the single coding exon and downstream of the stop codon in the 3' UTR mediate the deletion of a ~3.2 kb genomic segment ablating the coding region.	Due to their lack of B and T cells, these immune-compromised mice require enhanced husbandry practices. For breeding, transfer of wild-type B6 splenocytes helps restore temporary immune protection.
Il2rg	Elimination of innate lymphocytes and NK cells.	CRISPR guide targets in the *Il2rg* gene upstream of the transcription start site and downstream of the polyadenylation site excise a ~4.3 kb genomic segment encompassing the entire gene.	Loss of peripheral lymph nodes

Human genes are colored in green and mouse genes in blue. Acronym MAGIC for M-CSF, Rag1-/-, il2rg-/-, GMSCF, IL-6 and CD47 and MISTRG6v2 for M-CSF, IL6, SIRPA, THPO and GMSCF (version 2).

Legends for Supplementary Movies

Movie S1 (separate file). Human neutrophils from bone marrow of reconstituted MaGIC mice chemotaxing towards IL8. The EZ-TAXIScan (ECI Frontier, MIC-1000) was used to investigate chemotaxis of human neutrophils. Related to figure 4A. Movies are generated using Image J.

Movie S2 (separate file). Human neutrophils from fresh human bone marrow chemotaxing towards IL8. The EZ-TAXIScan (ECI Frontier, MIC-1000) was used to investigate chemotaxis of human neutrophils. Related to figure 4A. Movies are generated using Image J. This human reference data, analyzed side-by-side with human neutrophils from MaGIC mice, was previously published in Zheng and Sefik et al (2).

Legends for Datasets

Dataset S1 (separate file): Cluster identifying genes for neutrophils clusters as shown in Fig. 3 and S7. Gene names, average log fold change and adjusted p values are provided.

Dataset S2 (separate file): Genes that are enriched in spleen neutrophils compared to bone marrow and blood neutrophils. Gene names, average log fold change and adjusted p values are provided.

SI References

1. A. Rongvaux *et al.*, Development and function of human innate immune cells in a humanized mouse model. *Nat Biotechnol* **32**, 364-372 (2014).
2. Y. Zheng *et al.*, Human neutrophil development and functionality are enabled in a humanized mouse model. *Proc Natl Acad Sci U S A* **119**, e2121077119 (2022).
3. M. Chiorazzi *et al.*, Autologous humanized PDX modeling for immuno-oncology recapitulates features of the human tumor microenvironment. *J Immunother Cancer* **11** (2023).
4. H. Yu *et al.*, A novel humanized mouse model with significant improvement of class-switched, antigen-specific antibody production. *Blood* **129**, 959-969 (2017).
5. C. Xu *et al.*, Automatic cell-type harmonization and integration across Human Cell Atlas datasets. *Cell* **186**, 5876-5891 e5820 (2023).
6. C. Dominguez Conde *et al.*, Cross-tissue immune cell analysis reveals tissue-specific features in humans. *Science* **376**, eabl5197 (2022).
7. K. Street *et al.*, Slingshot: cell lineage and pseudotime inference for single-cell transcriptomics. *BMC Genomics* **19**, 477 (2018).
8. X. Xie *et al.*, Single-cell transcriptome profiling reveals neutrophil heterogeneity in homeostasis and infection. *Nat Immunol* **21**, 1119-1133 (2020).
9. B. J. van Raam, A. Drewniak, V. Groenewold, T. K. van den Berg, T. W. Kuijpers, Granulocyte colony-stimulating factor delays neutrophil apoptosis by inhibition of calpains upstream of caspase-3. *Blood* **112**, 2046-2054 (2008).
10. H. H. Woo, T. Baker, C. Laszlo, S. K. Chambers, Nucleolin mediates microRNA-directed CSF-1 mRNA deadenylation but increases translation of CSF-1 mRNA. *Mol Cell Proteomics* **12**, 1661-1677 (2013).
11. B. Stijlemans *et al.*, Development of a pHrodo-based assay for the assessment of in vitro and in vivo erythrophagocytosis during experimental trypanosomosis. *PLoS Negl Trop Dis* **9**, e0003561 (2015).
12. A. Rongvaux *et al.*, Development and function of human innate immune cells in a humanized mouse model. *Nature Biotechnology* **32**, 364-372 (2014).
13. J. M. Adrover *et al.*, A Neutrophil Timer Coordinates Immune Defense and Vascular Protection. *Immunity* **50**, 390-402 e310 (2019).
